# Wafer-scale and doping-tunable p-type semiconducting monolayer WSi_2_N_4_ film

**DOI:** 10.1093/nsr/nwag191

**Published:** 2026-03-26

**Authors:** Da-Yong Yang, Miaomiao Li, Lei Sun, Tianya Zhou, Jinmeng Tong, Chen Chen, Wenqin Zhao, Xuanya Liu, Huixuan Wang, Liping Zhao, Hanlin Li, Hui Li, Zhibo Liu, Xuefei Li, Shiqiao Qin, Mengjian Zhu, Chuan Xu, Wencai Ren

**Affiliations:** Shenyang National Laboratory for Materials Science, Institute of Metal Research, Chinese Academy of Sciences, Shenyang 110016, China; School of Materials Science and Engineering, University of Science and Technology of China, Shenyang 110016, China; College of Advanced Interdisciplinary Studies & Hunan Provincial Key Laboratory of Novel Nano-Optoelectronic Information Materials and Devices, National University of Defense Technology, Changsha 410073, China; Wuhan National High Magnetic Field Center, School of Integrated Circuits, Huazhong University of Science and Technology, Wuhan 430074, China; Shenyang National Laboratory for Materials Science, Institute of Metal Research, Chinese Academy of Sciences, Shenyang 110016, China; Shenyang National Laboratory for Materials Science, Institute of Metal Research, Chinese Academy of Sciences, Shenyang 110016, China; School of Materials Science and Engineering, University of Science and Technology of China, Shenyang 110016, China; Shenyang National Laboratory for Materials Science, Institute of Metal Research, Chinese Academy of Sciences, Shenyang 110016, China; School of Materials Science and Engineering, University of Science and Technology of China, Shenyang 110016, China; College of Advanced Interdisciplinary Studies & Hunan Provincial Key Laboratory of Novel Nano-Optoelectronic Information Materials and Devices, National University of Defense Technology, Changsha 410073, China; Shenyang National Laboratory for Materials Science, Institute of Metal Research, Chinese Academy of Sciences, Shenyang 110016, China; School of Materials Science and Engineering, University of Science and Technology of China, Shenyang 110016, China; Shenyang National Laboratory for Materials Science, Institute of Metal Research, Chinese Academy of Sciences, Shenyang 110016, China; School of Materials Science and Engineering, University of Science and Technology of China, Shenyang 110016, China; Shenyang National Laboratory for Materials Science, Institute of Metal Research, Chinese Academy of Sciences, Shenyang 110016, China; School of Materials Science and Engineering, University of Science and Technology of China, Shenyang 110016, China; Shenyang National Laboratory for Materials Science, Institute of Metal Research, Chinese Academy of Sciences, Shenyang 110016, China; School of Materials Science and Engineering, University of Science and Technology of China, Shenyang 110016, China; Shenyang National Laboratory for Materials Science, Institute of Metal Research, Chinese Academy of Sciences, Shenyang 110016, China; School of Materials Science and Engineering, University of Science and Technology of China, Shenyang 110016, China; Shenyang National Laboratory for Materials Science, Institute of Metal Research, Chinese Academy of Sciences, Shenyang 110016, China; School of Materials Science and Engineering, University of Science and Technology of China, Shenyang 110016, China; Wuhan National High Magnetic Field Center, School of Integrated Circuits, Huazhong University of Science and Technology, Wuhan 430074, China; College of Advanced Interdisciplinary Studies & Hunan Provincial Key Laboratory of Novel Nano-Optoelectronic Information Materials and Devices, National University of Defense Technology, Changsha 410073, China; College of Advanced Interdisciplinary Studies & Hunan Provincial Key Laboratory of Novel Nano-Optoelectronic Information Materials and Devices, National University of Defense Technology, Changsha 410073, China; Shenyang National Laboratory for Materials Science, Institute of Metal Research, Chinese Academy of Sciences, Shenyang 110016, China; School of Materials Science and Engineering, University of Science and Technology of China, Shenyang 110016, China; Shenyang National Laboratory for Materials Science, Institute of Metal Research, Chinese Academy of Sciences, Shenyang 110016, China; School of Materials Science and Engineering, University of Science and Technology of China, Shenyang 110016, China

**Keywords:** WSi_2_N_4_, two-dimensional semiconductors, CVD, wafer-scale growth, p-type doping

## Abstract

Monolayer WSi_2_N_4_ has been predicted to be a high-performance p-type semiconductor with high hole mobility, high on-state current density, high strength, and high thermal conductivity. Achieving large-area growth of monolayer WSi_2_N_4_ with controlled doping is a prerequisite for the scalable integration of device applications. However, only micrometer-sized monolayer WSi_2_N_4_ domains have been synthesized so far. Here, we report the growth of wafer-scale monolayer WSi_2_N_4_ films with submillimeter domains by chemical vapor deposition using a liquid Au/W bilayer as the growth substrate, with a growth rate three orders of magnitude higher than previously reported values. This method also enables efficient modulation of carrier doping concentration from 5.8 × 10^12^ cm^−2^ to 3.2 × 10^13^ cm^−2^ through *in situ* defect engineering. This doping-tunable monolayer WSi_2_N_4_ exhibits p-type semiconducting characteristics with a bandgap of ∼2.25 eV, achieving an on/off current ratio of 5.4 × 10^4^ at a low doping level and a high on-state current density (∼150 μA μm^−1^) and a low contact resistance (0.95 kΩ μm) for heavily doped material. Moreover, it has excellent stability and ultrahigh Young’s modulus (∼538 GPa) and strength (∼62 GPa). This work paves the way for the applications of monolayer WSi_2_N_4_ as a promising p-type channel material in 2D complementary metal-oxide-semiconductor integrated circuits.

## Teaser

Wafer-scale, doping-tunable monolayer WSi_2_N_4_ is synthesized, exhibiting p-type semiconducting behavior and establishing a platform for advanced 2D electronics.

## INTRODUCTION

Two-dimensional (2D) van der Waals (vdW) layered semiconductors have attracted tremendous interest as atomically thin channels that could facilitate continued transistor scaling thanks to their dangling-bond-free surface and little mobility variation with decreasing thickness [1–4]. So far, a wide range of n-type 2D semiconducting materials have been developed [1–4], such as MoS_2_ and MoSe_2_, but high-performance and stable p-type 2D semiconductors are scarce [5,6], which hinders the application of 2D semiconductors for positive-channel metal-oxide-semiconductor devices and complementary metal-oxide-semiconductor (CMOS) integrated circuits.

**Figure 1.**
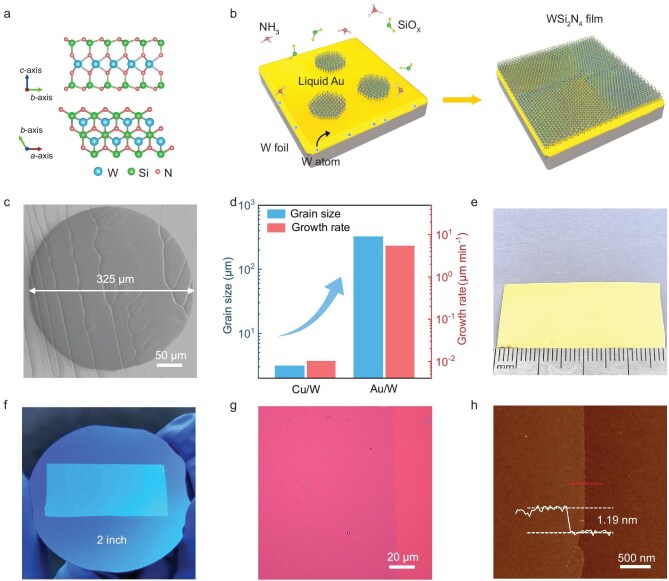
CVD growth of monolayer WSi_2_N_4_ crystals. (a) The cross-sectional (top) and in-plane (bottom) crystal structure of monolayer WSi_2_N_4_. (b) Schematic for the growth of monolayer WSi_2_N_4_ crystals. (c) Scanning electron microscopy (SEM) image of a circular WSi_2_N_4_ crystal with a lateral size of 325 μm. (d) Comparison of the domain sizes and growth rates of monolayer WSi_2_N_4_ grown on the liquid Au/W substrate and solid Cu/W substrate. (e, f) Photographs of a 3.6 × 1.8 cm^2^ monolayer WSi_2_N_4_ film grown on Au/W substrate (e) and after transferring onto a 2-inch SiO_2_/Si substrate (f). (g, h) Optical (g) and atomic force microscopy (AFM) (h) images of a monolayer WSi_2_N_4_ film transferred onto SiO_2_/Si substrate, showing no cracks or holes and uniform thickness of ∼1.19 nm.

The MoSi_2_N_4_ family is a newly emerging class of 2D vdW layered materials with a formula of MA_2_Z_4_ (M, A and Z denote an early transition metal, Si or Ge, and N, P or As, respectively) [7,8], which covers metals, semiconductors, superconductors, ferroelectrics and magnets [8–12]. Among them, centimeter-scale monolayer MoSi_2_N_4_ films have been synthesized by chemical vapor deposition (CVD) with a solid Cu/Mo bilayer as substrate [7], which show typical p-type semiconductor behavior with a bandgap of 1.94 eV, high breaking strength (∼66 GPa) and Young’s modulus (491 GPa), unusually high thermal conductivity (∼173 W m^−1^ K^−1^), and excellent stability [7,13]. However, for this CVD approach, metal atoms must diffuse through the solid Cu layer to react with the nitrogen atoms coming from the gas phase during growth. The slow diffusion rate of metal atoms in solids results in a low supply rate of metals, especially for W, which has the highest melting point among all known non-alloyed metals [14]. Thus, although monolayer WSi_2_N_4_ (Fig. 1a) has been predicted to be a high-performance p-type semiconductor with a larger bandgap and higher hole mobility, strength and thermal conductivity than those of monolayer MoSi_2_N_4_ as well as many other intriguing properties [15–19], only micrometer-sized monolayer domains could be obtained even after a long growth duration (5 h) [7].

Beyond wafer-scale growth, realizing the potential of monolayer WSi_2_N_4_ in complementary electronics requires well-controlled p-type conduction, which remains elusive for 2D semiconductors. Most current p-type doping strategies, including solvent intercalation [20], surface charge transfer [21], and metal oxide decoration [22], rely on post-growth modifications. These methods often suffer from limited spatial uniformity and insufficient long-term stability necessary for reliable device integration. Although *in situ* CVD modulation offers a promising alternative for scalable and reproducible carrier control on MoS_2_ and MoTe_2_ [23,24], its application to monolayer WSi_2_N_4_–including the modulation strategy, mechanism and its potential for tailoring electronic properties—remains unexplored. The lack of available wafer-scale materials and an efficient p-doping strategy significantly hinders the investigations of properties and device applications of monolayer WSi_2_N_4_.

Here, we report the growth of wafer-scale monolayer WSi_2_N_4_ films with submillimeter domains by CVD using a liquid Au/W bilayer as the growth substrate. The growth rate can reach 20 μm min^−1^, which is three orders of magnitude faster than that of monolayer WSi_2_N_4_ grown by CVD on a solid Cu/W substrate [7]. Furthermore, this method enables effective modulation of carrier density in monolayer WSi_2_N_4_ from 5.8 × 10^12^ cm^−2^ to 3.2 × 10^13^ cm^−2^ through *in situ* defect engineering. This doping-tunable monolayer WSi_2_N_4_ exhibits typical p-type semiconductor behavior with a bandgap of ∼2.25 eV, achieving an on/off current ratio of 5.4 × 10^4^ at a low doping level and an on-state current density up to ∼150 μA μm^−1^ and a contact resistance down to 0.95 kΩ μm for heavily doped material. Meanwhile, it shows ultrahigh strength (∼62 GPa) and Young’s modulus (∼538 GPa) as well as excellent stability.

## RESULTS AND DISCUSSION

Figure 1b illustrates the growth schematic of monolayer WSi_2_N_4_, where an Au foil sitting on the top of a W foil was used as the substrate, and NH_3_ gas and SiO_2_ plate as the precursors of N and Si, respectively. The Au/W substrate was first heated to a temperature above the melting point of Au (here, 1110°C), and then NH_3_ gas was introduced to initiate the growth of WSi_2_N_4_ (Fig. S1). First, the underlying W foil acted as the W source and a support for liquid Au, and the liquid Au layer not only provided a fast diffusion channel for W atoms [25] but also acted as a highly active catalyst [26,27]. Second, W atoms have a low solubility in liquid Au at 1110°C (∼0.32 atomic %) [28], avoiding the formation of WN_x_ due to an excessive supply of W atoms. Third, liquid Au spread well with a flat morphology on the surface of W foil in an NH_3_-containing atmosphere (Fig. S2), allowing for the growth of large-area WSi_2_N_4_. In contrast, liquid Cu exhibits poor wettability toward W foil in an NH_3_-containing atmosphere although it can spread well on the surface of W foil in a carbon-containing atmosphere [29], making it unsuitable for the growth of WSi_2_N_4_ film (Fig. S2). Thus, the liquid Au layer enabled the fast growth of large-area and high-quality uniform monolayer WSi_2_N_4_.

Figure 1c and d show that circular monolayer WSi_2_N_4_ domains with a lateral size of ∼325 μm were achieved on the liquid Au/W substrate within 60 min, yielding a growth rate of ∼5 μm min^−1^. Notably, the lateral size and growth rate of WSi_2_N_4_ were increased by ∼100 and ∼540 times, respectively, compared to those of WSi_2_N_4_ grown on a solid Cu/W substrate. Additionally, the morphology and growth rate of monolayer WSi_2_N_4_ domains can be effectively tuned by changing the growth atmosphere and temperature (Figs S3–S5). It is worth noting that no secondary phases except for WSi_2_N_4_ were formed in this wide growth window. By increasing the growth temperature to 1150°C, the growth rate reached 20 μm min^−1^ (Figs S6 and S7). However, the domains were randomly oriented due to the absence of an epitaxial relationship with the liquid Au substrate. After a growth period of 2 h, these isolated domains expanded and eventually merged into a wafer-scale polycrystalline WSi_2_N_4_ film (3.6 × 1.8 cm^2^), as shown in Fig. 1e and f. Notably, such WSi_2_N_4_ film is a uniform monolayer with a thickness of ∼1.17 nm without multilayer islands being formed even with extended growth time (Fig. 1g, h and Figs S8, S9), indicating a self-limiting surface adsorption growth behavior similar to graphene [30] and monolayer MoSi_2_N_4_ [7].

Different from the MoSi_2_N_4_ and WSi_2_N_4_ grown on Cu surface, there is a much weaker interfacial interaction between monolayer WSi_2_N_4_ and Au substrate (Fig. S10), which allowed for intact separation and transfer of monolayer WSi_2_N_4_ from Au substrate by using the electrochemical bubbling method [31] (Fig. 1e–h). Since this transfer approach relies on the bubbling-induced peeling force without Au etching being involved, no Au could be detected in the transferred monolayer WSi_2_N_4_ (Fig. S11). Moreover, the etching of WSi_2_N_4_ was avoided because no oxidizing etchant was used. As shown in Fig. S12, no cracks or holes were observed in an area of 100 × 100 nm^2^. In contrast, the WSi_2_N_4_ flakes transferred by etching Au were etched seriously with many holes and W clusters. The electrochemical bubbling transfer also enabled the repeated use of Au/W substrates for the growth of monolayer WSi_2_N_4_. As shown in Fig. S13, after 10 times of growth, both the substrate and WSi_2_N_4_ show no obvious difference with the original substrate and the first-grown WSi_2_N_4_ sample.

**Figure 2.**
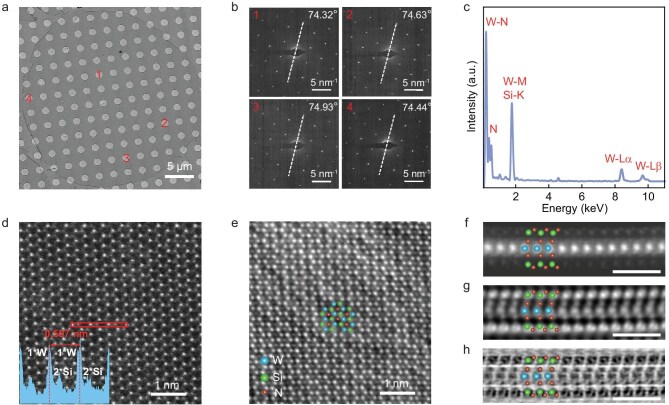
Structural characterization of monolayer WSi_2_N_4_ crystals. (a) Low-magnification TEM image of a circular monolayer WSi_2_N_4_ domain. (b) SAED patterns taken from the four regions marked by numbers in (a). The dashed lines represent the rotation angles (74.32, 74.63, 74.93 and 74.44°) relative to the horizontal line. (c) EDS spectrum taken from the monolayer WSi_2_N_4_ in (a). (d) Plan-view HAADF-STEM image of the monolayer WSi_2_N_4_ crystal. Inset is the intensity profile taken from the rectangular box, showing the lattice parameter of monolayer WSi_2_N_4_. (e) Plan-view iDPC-STEM image of the monolayer WSi_2_N_4_ crystal, showing that N atoms are located at the center of the six-membered ring composed of W and Si atoms. (f–h) HAADF- (f), iDPC- (g), and dDPC- (h) STEM images of the cross section of a monolayer WSi_2_N_4_ crystal. Scale bars: 1 nm in (f–h).

We further characterized the structure of transferred monolayer WSi_2_N_4_ domains by using transmission electron microscopy (TEM), energy-dispersive spectroscopy (EDS) and X-ray photoelectron spectroscopy (XPS). Figure 2a shows a circular monolayer WSi_2_N_4_ domain transferred onto a TEM grid. It features a clean surface, free of Au particles and polymer residues. The corresponding selected-area electron diffraction (SAED) patterns taken from four different regions show nearly identical crystallographic orientations (deviation smaller than ±0.5°), suggesting the single crystal nature of the circular WSi_2_N_4_ domains (Fig. 2b). This conclusion is extended to the triangular and hexagonal WSi_2_N_4_ domains by dark-field TEM imaging and SAED patterns, which jointly verify their single crystallinity (Figs S14–S16). The EDS profile shows that the crystal is composed of W, Si and N elements (Fig. 2c). The atomic-level high-angle annular dark field scanning TEM (HAADF-STEM) image shows a honeycomb lattice (Fig. 2d), where W and Si atoms are alternatively arranged based on the intensity profile along the (110) lattice plane (the inset in Fig. 2d). The extracted lattice parameter is ∼2.93 Å, in agreement with the reported structure of WSi_2_N_4_ [7]. The integrated differential phase contrast (iDPC) STEM image shows that N atoms are located at the center of the six-membered ring composed of W and Si atoms (Fig. 2e). The XPS spectra reveal the Si-N and W-N bonds in the crystal, with a W: Si: N atomic ratio approaching 1:2:4 (Fig. S17). The cross-sectional HAADF-, iDPC- and differentiated differential phase contrast (dDPC) STEM images reveal the septuple atomic layers of N-Si-N-W-N-Si-N (Fig. 2f-h), which is similar to that of MoSi_2_N_4_ [7].

**Figure 3.**
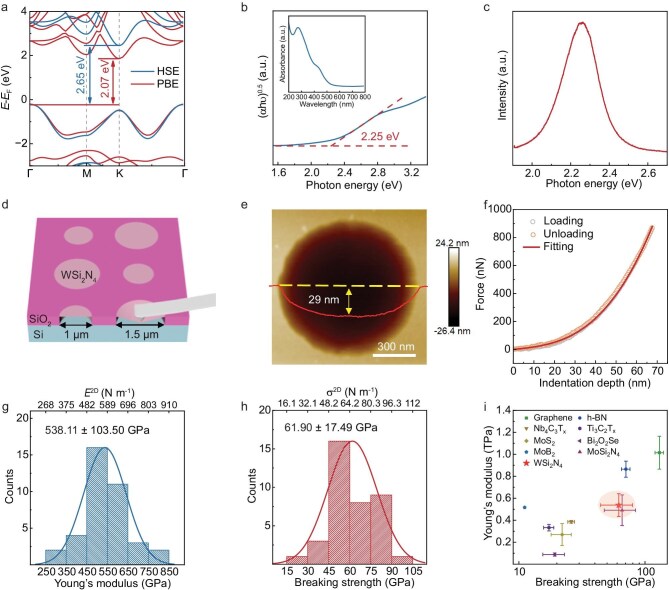
Optical and mechanical properties of monolayer WSi_2_N_4_. (a) Electronic band structure of monolayer WSi_2_N_4_ calculated with PBE (red lines) and HSE (blue lines). (b) Tauc plot extracted from the optical absorption spectrum (inset) of a monolayer WSi_2_N_4_ film, showing an optical bandgap of 2.25 eV. (c) The PL spectrum of a monolayer WSi_2_N_4_. (d) Schematic diagram of the nanoindentation method. (e) AFM topography of a suspended WSi_2_N_4_ monolayer over a hole; the height profile (red line) along the dashed line shows an indentation of ∼29 nm in the hole. (f) Typical force-displacement curves of a single-crystal WSi_2_N_4_ monolayer. The gray, orange, and red lines are the loading, unloading, and fitting curves, respectively. (g, h) Histograms of the Young’s modulus (g) and breaking strength (h) of monolayer single-crystal WSi_2_N_4_. Solid lines represent Gaussian fits to the data. (i) Comparison of Young’s modulus and breaking strength of monolayer WSi_2_N_4_ with those of typical 2D materials: graphene, *h*-BN, Nb_4_C_3_T_x_, Ti_3_C_2_T_x_, MoS_2_, Bi_2_O_2_Se, MoB_2_, MoSi_2_N_4_. See Table S1 for more details.

We calculated the electronic band structure of monolayer WSi_2_N_4_ using Perdew-Burke-Ernzerhof (PBE) functional and Heyd-Scuseria-Ernzerhof (HSE) functional. As shown in Fig. 3a, both calculations demonstrate that monolayer WSi_2_N_4_ is a semiconductor with an indirect bandgap (2.07 eV for PBE and 2.65 eV for HSE). We then measured the bandgap of monolayer WSi_2_N_4_ by using optical methods. The optical absorption spectrum of monolayer WSi_2_N_4_ film transferred onto a quartz substrate gives an optical bandgap of ∼2.25 eV (Fig. 3b). Figure 3c shows the photoluminescence (PL) spectrum of a monolayer WSi_2_N_4_ domain transferred on the SiO_2_/Si substrate, which exhibits a characteristic peak at 2.25 eV, consistent with the optical bandgap extracted from the optical absorption spectrum. These results indicate that monolayer WSi_2_N_4_ is a 2D semiconductor with a larger bandgap than that of monolayer MoSi_2_N_4_ (∼1.94 eV) [7].

Similar to monolayer MoSi_2_N_4_ [7], monolayer WSi_2_N_4_ shows excellent mechanical properties. We performed nanoindentation experiments to measure the Young’s modulus and breaking strength of monolayer WSi_2_N_4_. To do this, the monolayer WSi_2_N_4_ domains were transferred onto a SiO_2_/Si substrate with an array of holes (diameters of 1 and 1.5 μm) to create suspended membranes (Fig. 3d and e). We measured the force-displacement curves at the centers of the suspended WSi_2_N_4_ monolayer by using an AFM with a diamond tip. As shown in Fig. 3f, the identical loading and unloading curves demonstrate the elastic behavior of the membrane. On the basis of the thickness of monolayer WSi_2_N_4_ (1.07 nm) and testing data of 38 positions from different domains, the statistical Young’s modulus and breaking strength were extracted to be 538.11 ± 103.50 GPa and 61.9 ± 17.49 GPa, respectively (Fig. 3g and h), which are consistent with the theoretically calculated values (∼506 GPa and 55.5–59.2 GPa) [12]. It is worth noting that the Young’s modulus of monolayer WSi_2_N_4_ is higher than those of 2D materials beyond monolayer graphene and *h*-BN, such as MoB_2_, MoS_2_, Bi_2_O_2_Se, Ti_3_C_2_T_x_, Nb_4_C_3_T_x_, and MoSi_2_N_4_ (Fig. 3i).

**Figure 4.**
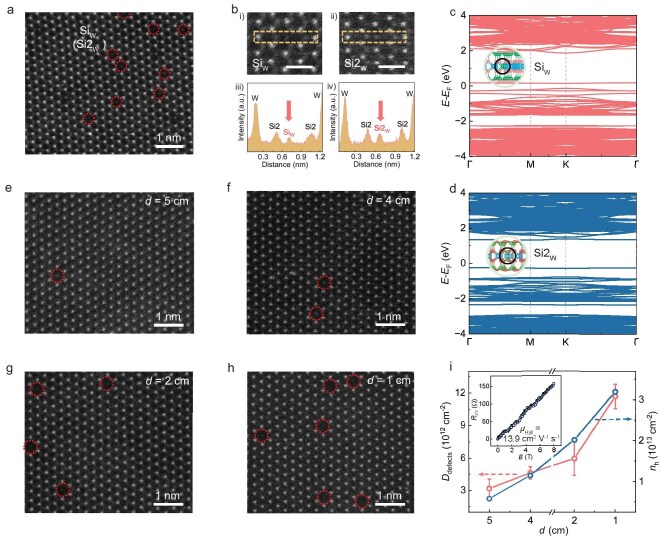
Origin and controlled modulation of p-type doping in monolayer WSi_2_N_4_. (a) HAADF-STEM image of a monolayer WSi_2_N_4_ with a high doping concentration, showing numerous Si_W_ and Si2_W_ antisite defects. (b) HAADF-STEM images and the corresponding intensity line profiles of Si_W_ (i, iii) and Si2_W_ (ii, iv) antisite defects. Scale bars in i and ii: 5 Å. (c, d) Calculated electronic band structures of monolayer WSi_2_N_4_ containing Si_W_ (c) and Si2_W_ (d) antisite defects. (e–h) HAADF-STEM images of monolayer WSi_2_N_4_ grown at different distances (*d*) between the quartz plate and the growth substrate. (i) Mean antisite defect density (*D*_defects_) (left axis) and hole concentration (*n*_h_) determined from Hall measurements (right axis) of monolayer WSi_2_N_4_ samples that were grown at different *d*. Inset: measured Hall resistance as a function of applied perpendicular magnetic field (*B*), yielding a Hall mobility of 13.9 cm^2^ V^−1^ s^−1^.

Previous theoretical calculations have shown that Si_Mo_ antisite defects exhibit the lowest formation energy among all point defects in MoSi_2_N_4_, leading to p-type doping [32]. Correspondingly, our extensive HAADF-STEM characterizations identified high-density Si_W_ and Si2_W_ antisite defects in our monolayer WSi_2_N_4_ (Fig. 4a and b), indicating their facile formation during CVD growth. We then studied the influence of Si_W_ and Si2_W_ antisite defects on the electronic band structure of WSi_2_N_4_ by first principles calculations. Similar to the case of monolayer MoSi_2_N_4_ [32], our results indicate that both Si_W_ and Si2_W_ antisite defects induce p-type doping in monolayer WSi_2_N_4_. Specifically, the Si_W_ antisite defect shifts the Fermi level down into the valence band, while the Si2_W_ antisite defect shifts the Fermi level close to the valence band edge (Fig. 4c and d, Fig. S18).

To modulate the carrier doping in monolayer WSi_2_N_4_, we developed a method to control the densities of Si_W_ and Si2_W_ antisite defects (*D*_defects_). We found that *D*_defects_ can be effectively tuned by adjusting the supply of Si source. Specifically, we reduced the Si feeding by increasing the distance (*d*) between the quartz plate and the growth substrate (Fig. S19). HAADF-STEM images show a rise in *D*_defects_ with decreasing *d* (Fig. S20). Quantitative analysis based on extensive HAADF-STEM imaging determined *D*_defects_ to be (1.17 ± 0.11) × 10^13^, (5.94 ± 1.55) × 10^12^, (4.61 ± 0.57) × 10^12^, and (3.18 ± 0.86) × 10^12^ cm^−2^ at *d* = 1, 2, 4, and 5 cm, respectively (Fig. 4e–i). Hall effect measurements were used to extract the corresponding hole concentrations (*n*_h_) (Fig. S21). The linear, positive slopes of *R*_XY_-*B* plots confirm hole-dominated transport, yielding a Hall mobility of ∼13.9 cm^2^ V^−1^ s^−1^ (Fig. 4i, inset). Notably, *n*_h_ increases from 5.8 × 10^12^ cm^−2^ to 3.2 × 10^13^ cm^−2^ as *D*_defects_ rises from (3.18 ± 0.86) × 10^12^ cm^−2^ to (1.17 ± 0.11) × 10^13^ cm^−2^ (Fig. 4i). Raman mapping on monolayer WSi_2_N_4_ films with varying *n*_h_ reveals spatially uniform peak positions that systematically shift with doping concentration (Figs S22 and S23), consistent with the doping-dependent core-level shifts observed in XPS (Fig. S24). The consistent shifts in both Raman and XPS peaks jointly demonstrate effective *in situ* modulation of hole concentration through controlled defect engineering during growth.

**Figure 5.**
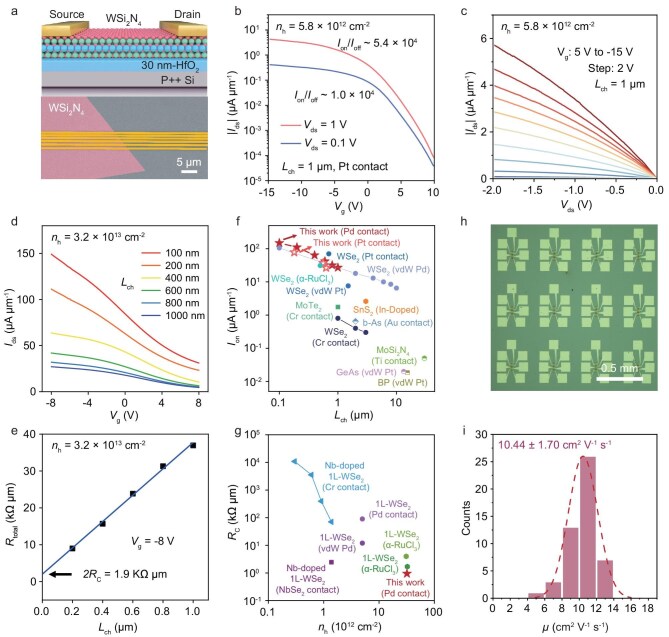
Device performance of monolayer WSi_2_N_4_ FETs. (a) Schematic (top) and SEM image (bottom) of monolayer WSi_2_N_4_ FET on HfO_2_ dielectric. (b, c) Transfer (b) and output (c) characteristics of monolayer WSi_2_N_4_ FETs with a doping concentration of 5.8 × 10^12^ cm^−2^. The channel length and width are 1 μm and 1.4 μm, respectively. (d) The transfer curves for monolayer WSi_2_N_4_ FETs with a doping concentration of 3.2 × 10^13^ cm^−2^, measured across channel lengths from 100 nm to 1 μm. (e) Extracted contact resistance of monolayer WSi_2_N_4_ using the TLM method. (f, g) Comparison of on-state current densities (f) and contact resistances (g) of monolayer WSi_2_N_4_ FETs with those of reported p-type monolayer semiconductor-based FETs. See Table S4 and Table S5 for more details. (h) Optical image of a fabricated FET array. (i) Statistical distribution of field-effect mobility.

We fabricated field-effect transistors (FETs) on a HfO_2_/Si substrate using monolayer WSi_2_N_4_ with different doping concentrations (Fig. 5a). Pt was selected as the source/drain electrode due to its higher work function (∼5.65 eV), which provides favorable energy level alignment with the VBM of WSi_2_N_4_ (∼5.71 eV) to facilitate hole injection (Fig. S25). For monolayer WSi_2_N_4_ with a relatively low doping concentration of 5.8 × 10^12^ cm^−2^, the FET with a 1-μm channel length demonstrates typical p-type transistor behavior. The transfer characteristics (Fig. 5b), measured at *V*_ds_ = 1 V with *V*_g_ swept from −15 V to 10 V, show an on-state current density of ∼4.5 μA μm^−1^ (at *V*_g_ = −15 V) and an on/off ratio of 5.4 × 10^4^. The linear output characteristics (*I*_ds_–*V*_ds_) indicate excellent p-type ohmic contact (Fig. 5c), confirming a low Schottky barrier at the Pt/WSi_2_N_4_ contact interface (Fig. S26, Table S2). We further integrated the p-type WSi_2_N_4_ with CVD-grown monolayer MoS_2_ to construct a 2D CMOS inverter (Fig. S27). The static voltage transfer characteristic demonstrates signal-inverting behavior, with a peak voltage gain of ∼5 at *V*_DD_ = 3 V, demonstrating the potential of integrating wafer-scale p-type monolayer WSi_2_N_4_ with other n-type 2D semiconductors for advanced logic circuits.

By significantly increasing the hole concentration, the resulting heavily doped monolayer WSi_2_N_4_ enables ultralow contact resistance, which in turn leads to a remarkably high on-state current density. This addresses a major challenge in p-type 2D semiconductors, where devices often suffer from highly resistive contacts, resulting in current densities at least an order of magnitude lower than those of n-type channel materials such as MoS_2_ and InSe [33–35]. As shown in Fig. S28, the contact resistance of devices monotonically decreases as increasing the doping concentration of WSi_2_N_4_. For a 100 nm short-channel transistor with a doping concentration of 3.2 × 10^13^ cm^−2^, we achieved an on-state current density of ∼150 μA μm^−1^ at *V*_ds_ = 1 V (Fig. 5d), with a corresponding contact resistance (*R*_C_) of ∼0.95 kΩ μm as extracted by the transfer length method (TLM) (Fig. 5e). Notably, these values rank among the best reported for p-type monolayer semiconductor FETs with similar channel length and measurement conditions (Fig. 5f and g, Tables S4 and S5).

Beyond a single device, our WSi_2_N_4_ films enable the batch fabrication of FET arrays (Fig. 5h). As an example, we fabricated FET arrays (channel length, 10 μm) using a moderately doped monolayer WSi_2_N_4_ film (1.15 × 10^13^ cm^−2^). All FETs exhibit excellent uniformity, as evidenced by the narrow distribution of the field-effect mobility (10.44 ± 1.70 cm^2^ V^−1^ s^−1^), which is consistent with the Hall mobility (13.9 cm^2^ V^−1^ s^−1^) (Fig. 5i), and a stable on-state current density (5.5 ± 0.2 μA μm^−1^) (Fig. S29). These statistical results robustly confirm the high electronic homogeneity of our monolayer WSi_2_N_4_ film. Notably, while grain boundaries (GBs) can locally degrade mobility by introducing scattering and trap states (Fig. S30, Table S6), the constituent grains in our films are sufficiently large, minimizing GB density and preserving the overall uniformity and performance of our FETs.

Importantly, our monolayer WSi_2_N_4_ has excellent chemical and thermal stability. The PL spectra of monolayer WSi_2_N_4_ crystals remained almost unchanged after they were immersed in acetone, isopropanol and 1 M NaOH solution for 24 h (Fig. S31), or placed under ambient conditions for 6 months, in non-degassed deionized water for 1 week, and in 80°C non-degassed deionized water for 8 h (Fig. S32), or annealed at 300°C in Ar for 3 h (Fig. S33). In particular, as a p-type 2D semiconductor, monolayer WSi_2_N_4_ shows overwhelming superiority in stability over the typical p-type 2D semiconductor, monolayer WSe_2_. Compared to WSe_2_, the significantly higher formation energies of various defects in WSi_2_N_4_ make its degradation and oxidation more difficult (Table S7). As shown in Fig. S34, monolayer WSi_2_N_4_ crystals have no visible change after immersion in water, exposure to air, and annealing in air. In contrast, the high-quality monolayer WSe_2_ crystals grown by CVD [26] are seriously damaged or even completely disappear under the same conditions. Furthermore, FETs based on monolayer WSi_2_N_4_ show negligible performance degradation even after 3 months in ambient air, 10 min of immersion in deionized water, and over 22 sweep cycles (Fig. S35), confirming their outstanding environmental and operational stability for device applications.

## CONCLUSION

This work demonstrates the wafer-scale CVD growth of doping-tunable p-type monolayer WSi_2_N_4_ films, which paves the way for their applications in CMOS integrated circuits. More importantly, this CVD method using liquid Au as a growth substrate provides a general and highly efficient way for the fast growth of other MA_2_Z_4_ materials. For instance, we realized the fast growth of high-quality monolayer MoSi_2_N_4_ by using a liquid Au/Mo/Re substrate (Figs S36 and S37), where an ultrathin Mo film was deposited on Re foil to avoid excessive supply of Mo atoms as Mo has a much higher solubility in liquid Au than W. Monolayer MoSi_2_N_4_ crystals of ∼145 μm in lateral size were obtained in 30 min, corresponding to a growth rate of ∼4.83 μm min^−1^, which is about 8 times higher than that of monolayer MoSi_2_N_4_ crystals grown on solid Cu/Mo substrate as reported previously (∼0.64 μm min^−1^) [7].

## Supplementary Material

nwag191_Supplemental_File
