# Supplementary material for: Wafer-scale and doping-tunable p-type semiconducting monolayer WSi2N4 film
Source: Natl Sci Rev. 2026 Mar 26;13(10):nwag191. doi: 10.1093/nsr/nwag191 (PMC13211991; doi:10.1093/nsr/nwag191)
Supplement: nwag191_Supplemental_File [file nwag191_supplemental_file.pdf]

Supplementary information for

Wafer-scale and doping-tunable p-type semiconducting monolayer WSi_2N_4 film

Da-Yong Yang[1,2,†], Miaomiao Li[3,†], Lei Sun[4], Tianya Zhou[1], Jinmeng Tong[1,2], Chen Chen[1,2], Wenqin Zhao[3], Xuanya Liu[1,2], Huixuan Wang[1,2], Liping Zhao[1,2], Hanlin Li[1,2], Hui Li[1,2], Zhibo Liu[1,2], Xuefei Li[4], Shiqiao Qin[3], Mengjian Zhu[3,*], Chuan Xu[1,2,*] and Wencai Ren[1,2,*]

[1]Shenyang National Laboratory for Materials Science, Institute of Metal Research, Chinese Academy of Sciences, Shenyang 110016, China;

[2]School of Materials Science and Engineering, University of Science and Technology of China, Shenyang 110016, China;

[3]College of Advanced Interdisciplinary Studies & Hunan Provincial Key Laboratory of Novel Nano-Optoelectronic Information Materials and Devices, National University of Defense Technology, Changsha, Hunan 410073, China;

[4]Wuhan National High Magnetic Field Center, School of Integrated Circuits, Huazhong University of Science and Technology, Wuhan 430074, China

***Corresponding authors**. E-mails: wcren@imr.ac.cn; xuc@imr.ac.cn; zhumengjian11@nudt.edu.cn

†Equally contributed to this work.

METHODS

CVD growth of monolayer WSi_2N_4 and $MoSi_2N_4$ on liquid Au. A piece of Au foil (99.99 wt%, 10 μm thick, ZhongNuo Advanced Material (Beijing) Technology Co., Ltd) was cut into 3.6 × 1.8 cm^2 pieces and placed on the top of a piece of W foil (99.95 wt%, 50 μm thick, Alfa Aesar) of the same size as the growth substrate. The Au/W bilayer substrate was placed in a pyrolytic-BN tube to shield the influence of Si atoms coming from the quartz tube. A quartz plate (500 μm thick, ~14 × 14 mm^2 in size, Shenyang KeJing Auto-Instrument Co., Ltd) or a pure Si plate (650 μm thick, ~14 × 14 mm^2 in size, Shenyang KeJing Auto-Instrument Co., Ltd) served as the silicon source. The silicon source concentration during the growth of WSi_2N_4 was regulated by varying the distance (d) between the quartz plate (or a pure Si plate) and the growth substrate. The Au/W substrate was then heated to 1080 – 1150 °C in a horizontal tube furnace under an ultrahigh-purity hydrogen (H_2) atmosphere (99.999% purity, 300 sccm, Dalian Special Gases Co., Ltd). After annealing for 15 – 30 min to ensure enough W atoms to diffuse through the liquid Au to the Au surface, 6 – 8 sccm NH_3 (99.0% purity, Dalian Special Gases Co., Ltd) flow was introduced into the reaction tube at ambient pressure to initiate the growth of monolayer WSi_2N_4 crystals. After a certain reaction time (15 – 120 min), the substrate was quickly moved from the high-temperature zone under H_2 atmosphere to stop the growth. For the growth of monolayer $MoSi_2N_4$, a Mo film deposited on a Re foil (99.97 wt%, 25 μm thick, Alfa Aesar) was used as Mo source, and the growth process was similar to that used for the growth of monolayer WSi_2N_4.

Bubbling transfer of monolayer WSi_2N_4 from Au/W substrate. A protective layer of polymethyl methacrylate (PMMA, 950 kDa molecular weight, Sigma, 4 wt% in ethyl lactate) was spin-coated on the surface of the WSi_2N_4 grown on Au/W substrate at 2500 r.p.m. for 60 s and heated at 180 °C for 20 min. Then, the PMMA/WSi_2N_4/Au/W stack, Pt foil and 1 M NaOH solution were used as the cathode, anode and electrolyte, respectively. After the PMMA/WSi_2N_4 stack was separated from the Au/W substrate by H_2 bubbles produced by water electrolysis at a current of 200 mA, it was collected onto the target substrate, and finally the coated PMMA was removed in hot acetone.

Structure characterizations. The morphology of WSi_2N_4 was characterized using an optical microscope (Nikon LV100D) and SEM (Verios G4 UC, acceleration voltage of 10 kV). The thickness was measured using an AFM (MultiMode 8, Bruker, Inc.). XPS and UPS measurements were conducted on an ESCALAB 250 spectrometer using monochromatic Al Kα radiation (1486.6 eV). Optical absorption spectra were measured by UV–vis–IR diffuse reflectance spectrophotometer (Jasco V-770). Raman and PL spectra were collected with a confocal Raman spectrometer (Horiba, LabRAM Odyssey) using a 325-nm laser as the excitation source, respectively. The TEM morphology, SAED and dark-field TEM characterizations were performed on a TEM (FEI Talos F200x) operating at 200 kV. The HAADF-, iDPC-, dDPC-STEM and EDS measurements were performed in STEM mode at 300 kV on a FEI Titan Cube Themis G2 300 instrument equipped with a high-brightness field-emission gun (X-FEG), double spherical aberration corrector, and a monochromator. The cross-sectional STEM samples were fabricated by focused ion beam (FIB) cutting on FEI Helios NanoLab

DualBeam 235.

First principles calculations on the electronic band structure. First-principles calculations were performed by using the Vienna ab initio simulation package (VASP)[1-3]. The projector augmented wave[4] method and generalized gradient approximation with the Perdew–Burke–Ernzerhof exchange-correlation functional (GGA-PBE)[2,5] were utilized. The cutoff energy for plane-wave expansion was set to 600 eV. The 5 × 5 × 1 WSi_2N_4 supercell was constructed for antisite defects, and a vacuum of 20 Å between layers was set up to minimize the interactions between the periodic layers along the c-axis direction. The unit cell and supercells were fully relaxed with 15 × 15 × 1 and 2 × 2 × 1 k-mesh until the energy and force converged below 10^{-8} eV and 0.02 eV $Å^{-1}$, respectively. To address the potential underestimation of the bandgap within GGA-PBE, the nonlocal HSE06 hybrid functional was used with 6 × 6 × 1 k-mesh and 10^{-8} eV energy convergence for electronic structure calculations.

Mechanical property measurements and data analysis. We measured the force curve of monolayer WSi_2N_4 as reported previously[7]. The monolayer WSi_2N_4 domains (>30 μm) were transferred onto a SiO_2 (285 nm thick)/Si substrate with circular holes of 1 μm and 1.5 μm in diameter to create a suspended membrane. The force-displacement curves were measured by an AFM (MultiMode 8, Bruker, Inc.), where a diamond tip (AD-150-NM, Adama) with a radius (*R*) of 9.58 nm and cantilever spring constant of ~153.0 N m^{-1} was used. During the indentation experiments, the sample was moved up vertically to make contact with the tip. In this procedure, two factors were measured,

the change of cantilever deflection and z-piezo extension. The force F was obtained through multiplying the cantilever deflection by the cantilever spring constant. The real displacement δ was calculated by subtracting the cantilever deflection from the z-piezo extension. A set of force-displacement curves were finally acquired. Then the curves were fitted by using the formula

$$F = \sigma_0^{2D}(\pi\delta) + E^{2D}\frac{q^3\delta^3}{a^2} \tag{1}$$

Where σ_0^{2D} denotes prestress in the membrane, δ is the real displacement, E^{2D} is the 2D elastic modulus, and a is the radius of the hole, and q is a dimensionless constant, equal to $1/(1.05–0.15v–0.16v^2)$, in which v denotes the Poisson's ratio of the membrane. Here, we took $v = 0.280$ by first-principles calculations, thus $q = 1.005$. According to the calculated 2D elastic modulus E^{2D}, measured breaking force F_m and tip radius R, the 2D breaking strength (σ_{max}^{2D}) of monolayer WSi_2N_4 was modeled in the linear elastic regime by using the expression

$$\sigma_{max}^{2D} = \left(\frac{F_m E^{2D}}{4\pi R}\right)^{\frac{1}{2}} \tag{2}$$

FET device fabrication and electrical property measurements. The monolayer WSi_2N_4 was transferred onto SiO_2 (300 nm)/Si and HfO_2 (30 nm)/Si substrates to fabricate Hall bar devices and FETs, respectively. For WSi_2N_4 FETs, the HfO_2 layer was prepared by atomic layer deposition (ALD) technique and it exhibited a dielectric constant of ~10. After spin-coating PMMA at 4000 rpm for 60 s, the samples were dried at 175 °C for 5 min. Then, the source and drain electrodes were patterned using electron-beam lithography (EBL), followed by the deposition of 10 nm-thick Pt and 20-

nm-thick Au (for weakly doped WSi_2N_4) or 10 nm-thick Pd and 50-nm-thick Au (for medium and heavily- doped WSi_2N_4) in sequence via electron beam evaporation system. The resulting monolayer WSi_2N_4 FETs were further thermally annealed in ultra-high vacuum (~ 10^{-8} Torr) at 220 °C for 3 h. The I_{ds}-V_{ds} and I_{ds}-V_g characteristics of monolayer WSi_2N_4 FETs were measured by a probe station, which was equipped with a precision source meter in a high vacuum chamber of 10^{-8} Torr, at temperatures ranging from 300 to 10 K. Hall measurements up to 8 T were performed in a cryogenic system. The magnetic field was applied perpendicular to the sample surface. An alternating current (AC) lock-in technique was used to measure the Hall devices. The AC source/drain current was 1 μA for all measurements.

Supplementary figures and tables

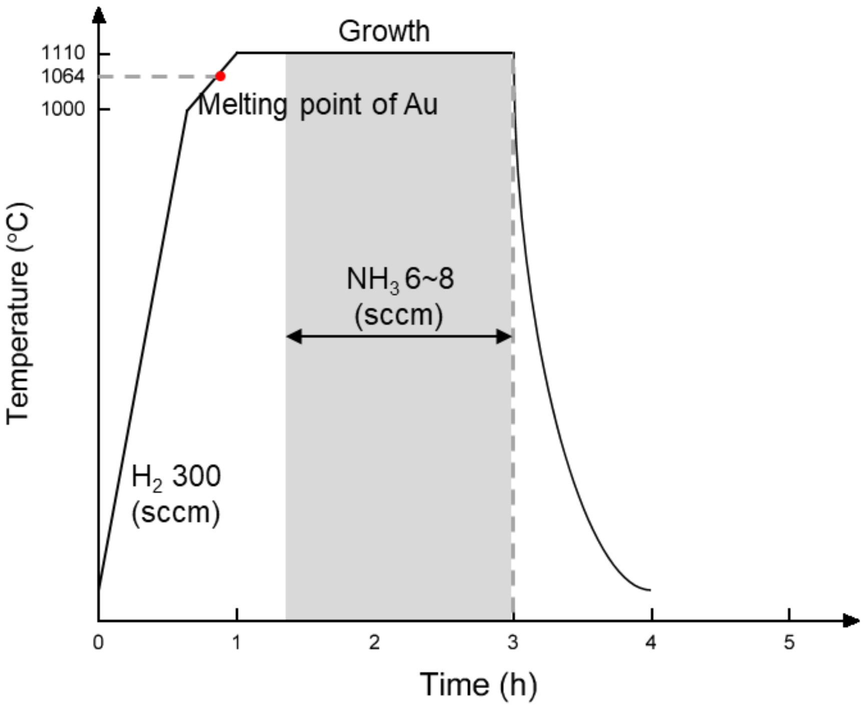

Figure S1. Typical heating procedure for the growth of monolayer WSi_2N_4.

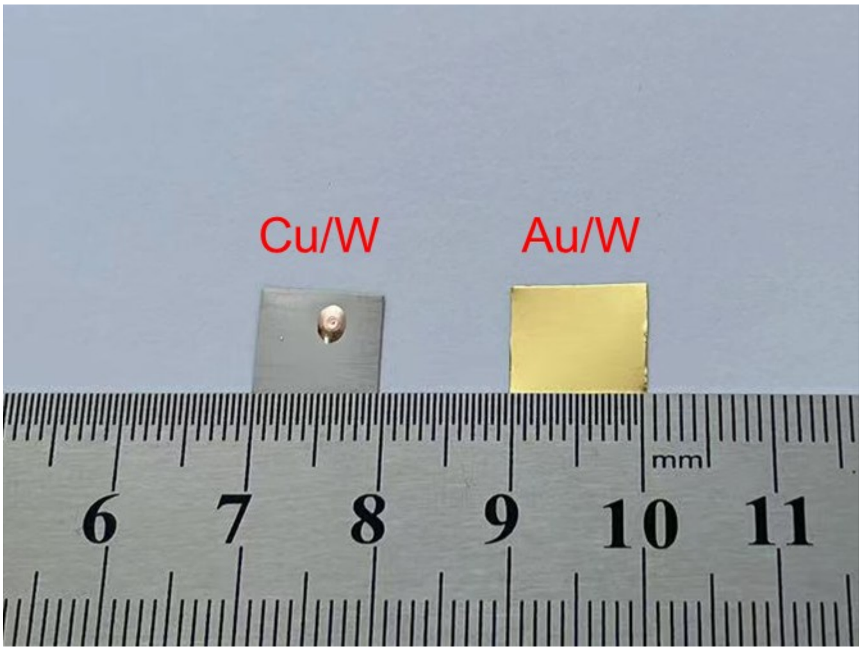

Figure S2. Photograph of the resolidified liquid Cu/W and liquid Au/W substrates after CVD growth, showing that the wettability of liquid Au on tungsten is much better than that of liquid Cu on tungsten in an ammonia-containing atmosphere.

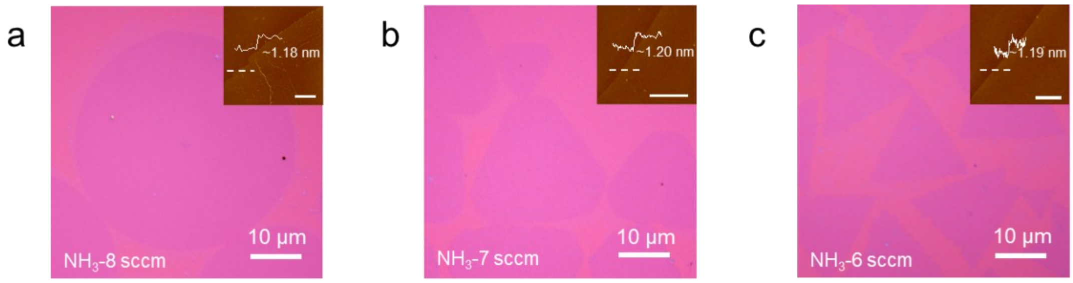

Figure S3. Optical images of monolayer WSi_2N_4 domains transferred onto SiO_2/Si substrates, which were grown under different NH_3 flow rates at 1110 °C for 15 min. The NH_3 flow rates for the growth of monolayer WSi_2N_4 domains in (a-c) are 8, 7 and 6 sccm, respectively. The insets in (a-c) are the corresponding AFM images of WSi_2N_4 domains, showing their thicknesses remain ~1.17 nm under different NH_3 flow rates. Scale bars in the insets: 2 μm in (a)**,** 500 nm in (b, c).

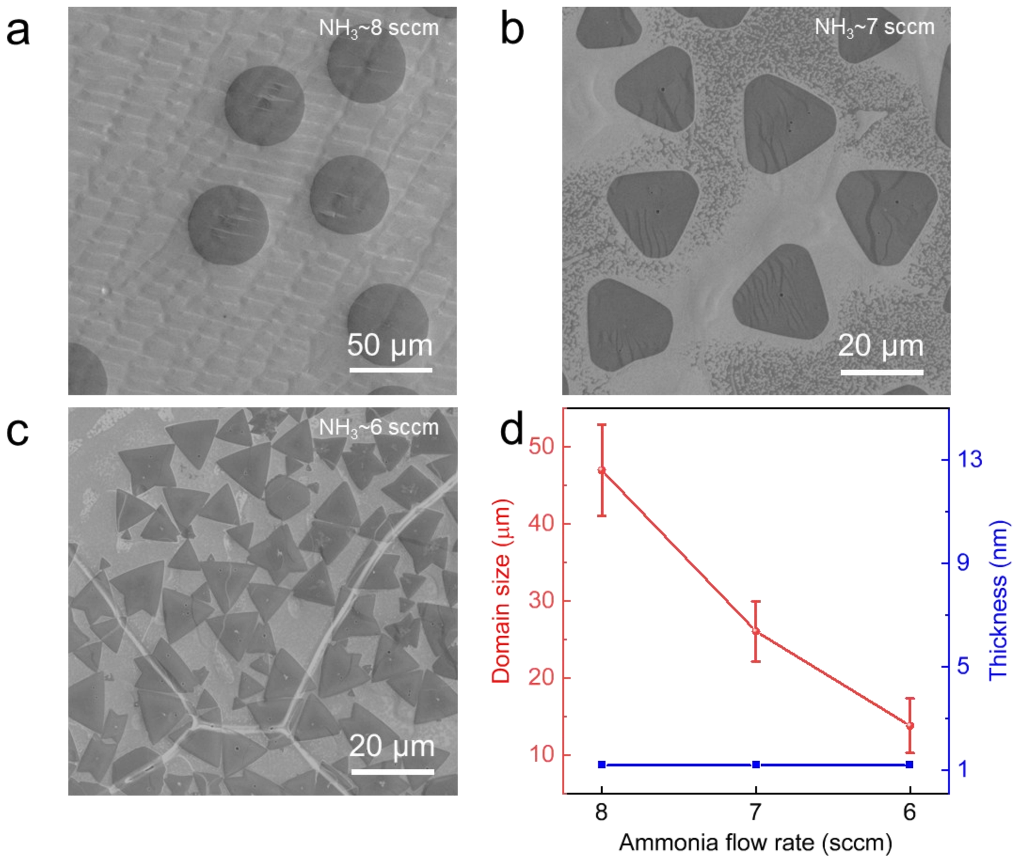

Figure S4. (a-c) SEM images of monolayer WSi_2N_4 domains grown at 1110 °C for 15 min under 8 (a), 7 (b) and 6 (c) sccm NH_3. (d) The lateral size and thickness of monolayer WSi_2N_4 domains as a function of NH_3 flow rate.

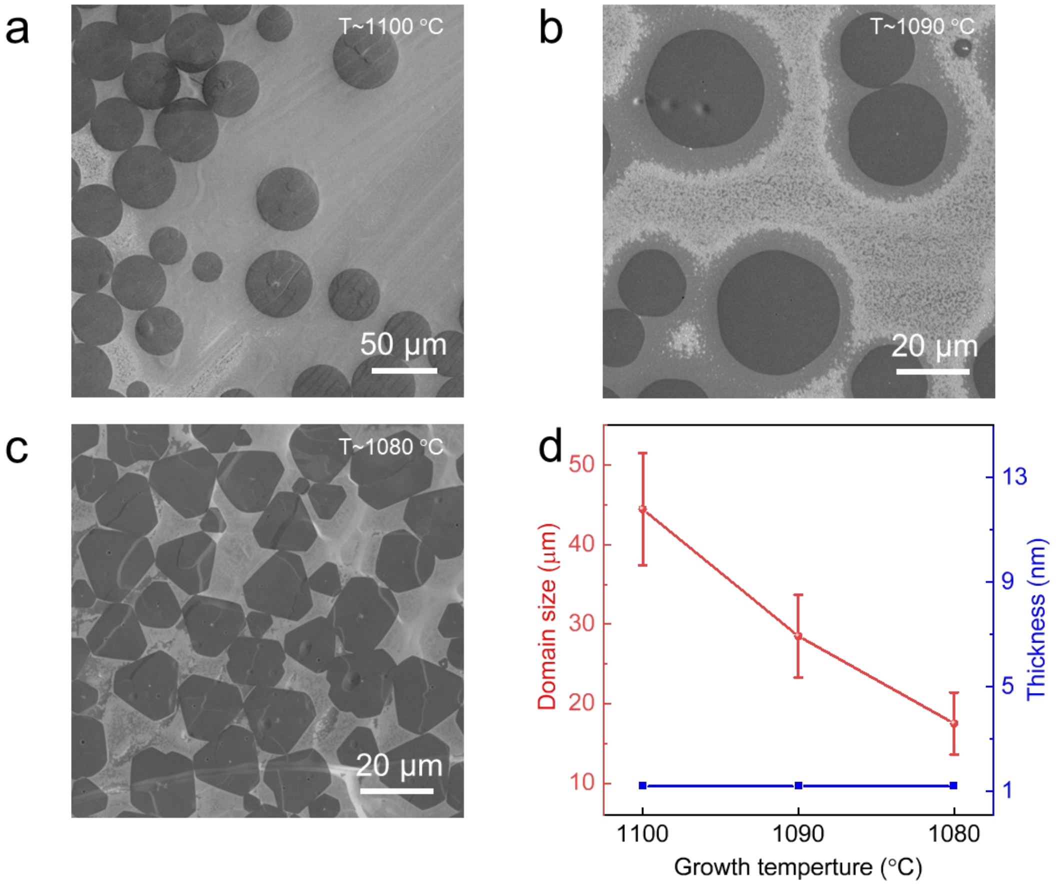

Figure S5. (a-c) SEM images of monolayer WSi_2N_4 domains grown under 8 sccm NH_3 for 15 min at 1100 °C (a), 1090 °C (b), and 1080 °C (c). (d) The lateral size and thickness of monolayer WSi_2N_4 domains as a function of growth temperature.

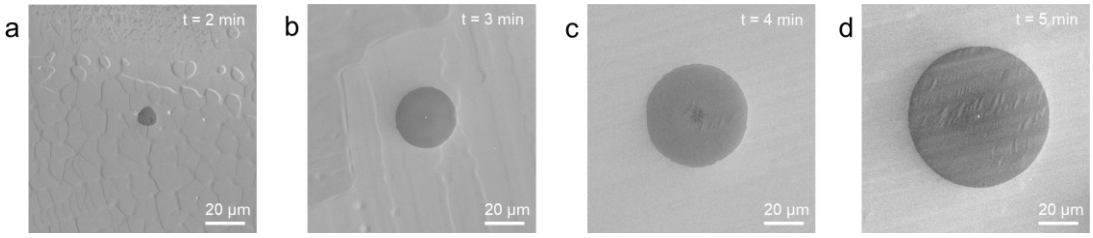

Figure S6. SEM images of monolayer WSi_2N_4 domains grown on Au/W substrate at 1150 °C for 2 min (a), 3 min (b), 4 min (c), and 5 min (d).

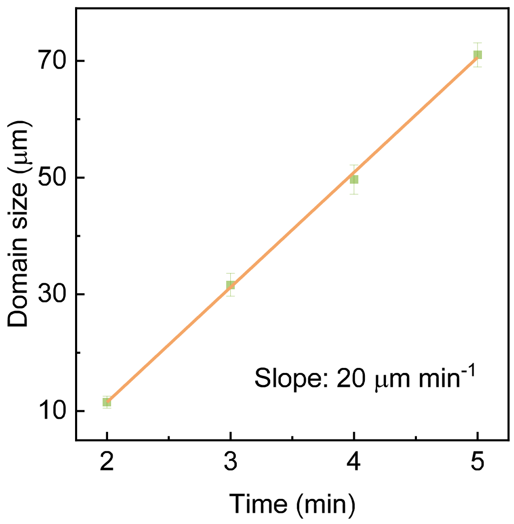

Figure S7. The size of WSi_2N_4 domains as a function of growth time with a linear fit at 1150 °C. The slope indicates a high growth rate of ~20 μm min^{-1}.

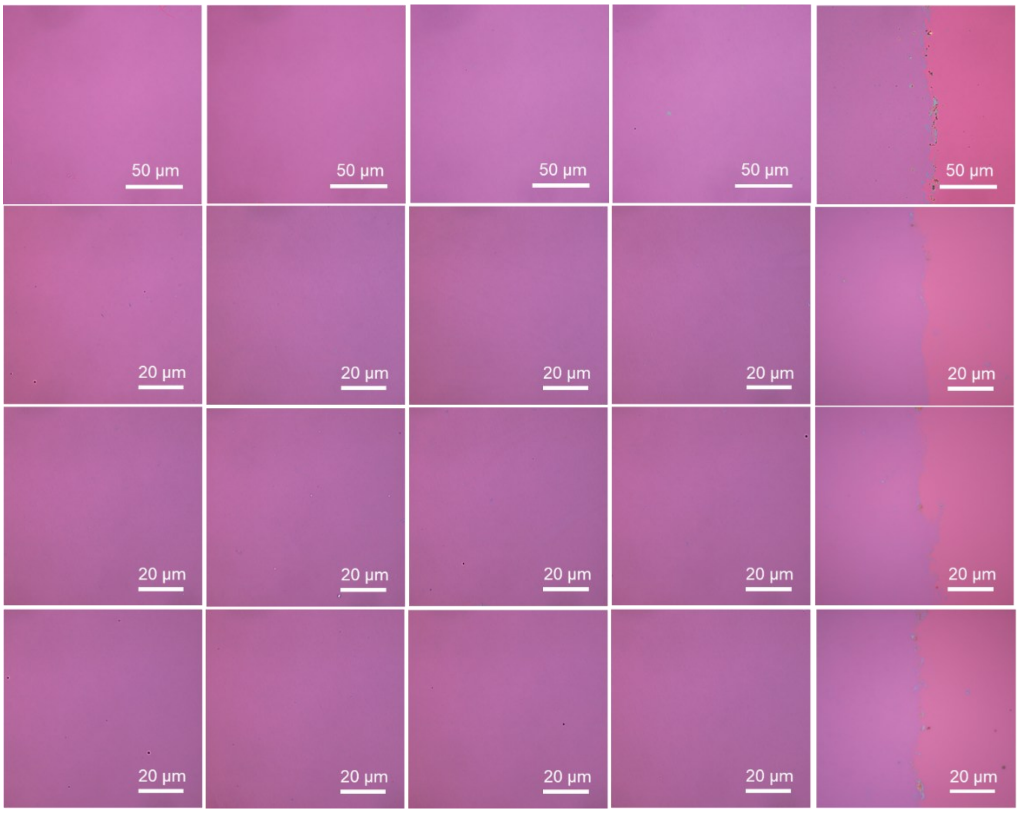

Figure S8. Optical images of randomly selected regions in a monolayer WSi_2N_4 film transferred on SiO_2/Si substrate.

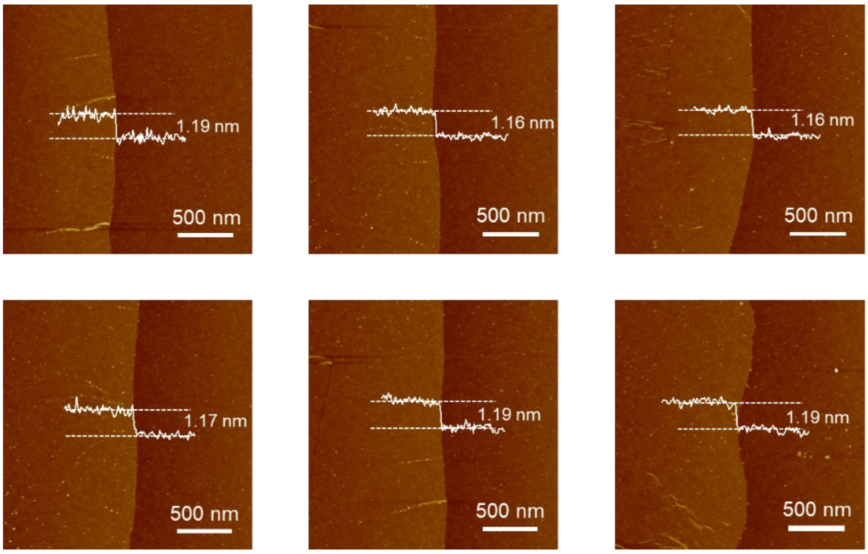

Figure S9. AFM images taken from different locations, showing the good uniformity of the monolayer WSi_2N_4 film transferred on SiO_2/Si substrates.

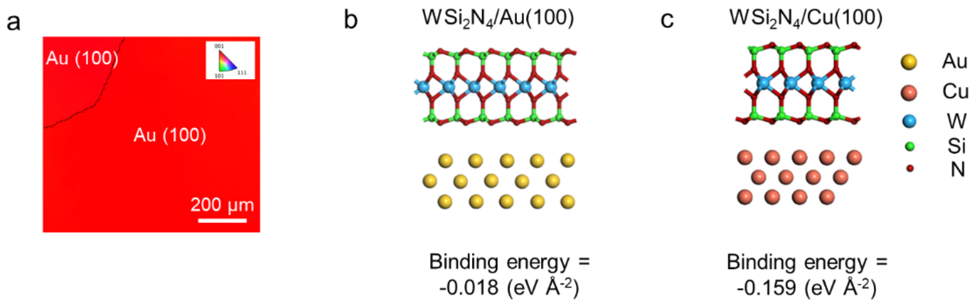

Figure S10. Comparison of the interfacial interactions of WSi_2N_4/Au (100) and WSi_2N_4/Cu (100). (a) EBSD IPF map of resolidified Au on the W foil after WSi_2N_4 growth. (b, c) Calculated binding energies between WSi_2N_4 and Au (100) (b) and between WSi_2N_4 and Cu (100) (c). Note that the WSi_2N_4/Au (100) has a weaker interfacial interaction than WSi_2N_4/Cu (100).

First-principles calculations were performed by using the Vienna ab initio simulation package (VASP)[1-3] based on DFT[6]. The projected augmented wave (PAW) method[4] and generalized gradient approximation with the Perdew-Burke-Ernzerh exchange-correlation functional (GGA-PBE)[2,5] and the DFT-D3 method[8] (Grimme scheme with zero damping) were used. The plane-wave cutoff energy of 500 eV was used with a 15 × 15 × 1 k-mesh. A large vacuum space was set to 20 Å to avoid the interactions between the neighboring layers. The monolayer WSi_2N_4, Au (100), Cu (100) were relaxed until the energies and forces converged below 10^{-8} eV and 0.02 eV $Å^{-1}$, respectively.

The lattice match (lattice mismatch less than 5%) and convergence criterion of the heterostructures are shown as follows. 5 × 1 ($\sqrt{3}$ × 1) WSi_2N_4 matches 3 × 1 Au (100). The WSi_2N_4/Au (100) was relaxed with a 2 × 9 × 1 k-mesh until the energy and force converged below 10^{-7} eV and 0.05 eV $Å^{-1}$, respectively. 7 × 1 ($\sqrt{3}$ × 1) WSi_2N_4 matches 8 × 2 Cu (100). The WSi_2N_4/Cu (100) was relaxed with a 5 × 1 × 1 k-mesh

until the energy and force converged below 10^{-6} eV and 0.1 eV $Å^{-1}$, respectively. The binding energy E_B is defined as follows

$$E_B = \frac{E_{Heter} - E_{WSN} - E_{Metal}}{N}$$

Where E_{Heter}, E_{WSN}, E_{Metal} and N are the energies of heterostructures, WSi_2N_4 supercell, metal (100) supercell and the number of WSi_2N_4 unit cells, respectively.

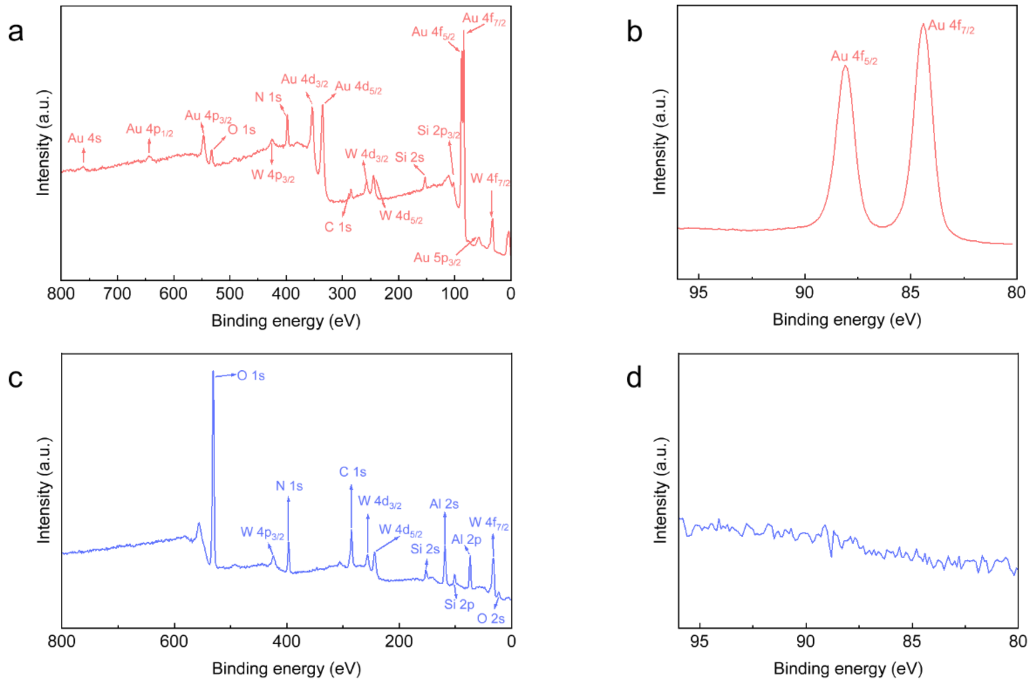

Figure S11. XPS characterization of as-grown and transferred monolayer WSi_2N_4. (a, b) Survey (a) and Au 4f (b) XPS spectra of a monolayer WSi_2N_4 film grown on the Au/W substrate. (c, d) Survey (c) and Au 4f (d) XPS spectra of a monolayer WSi_2N_4 film transferred onto a sapphire substrate. There is no detectable Au 4f peaks in WSi_2N_4 transferred on sapphire substrate.

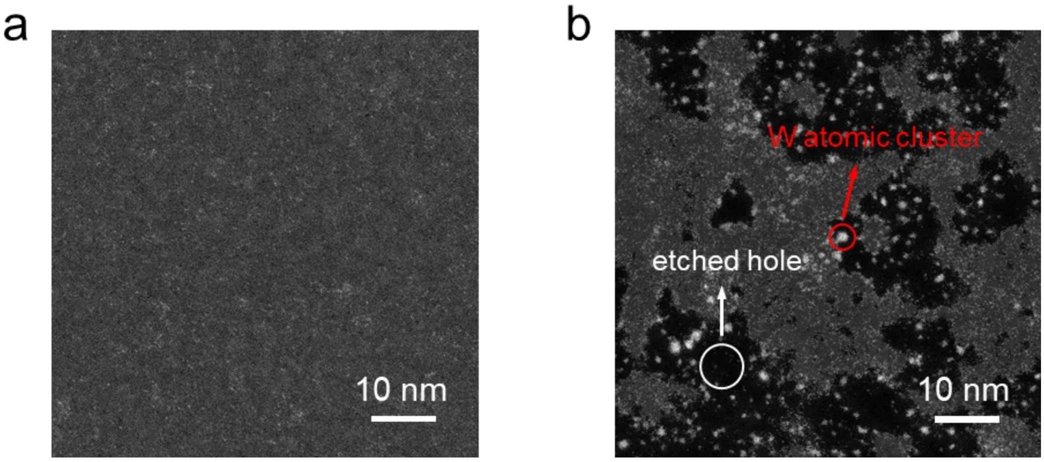

Figure S12. The low-magnification HAADF-STEM images of monolayer WSi_2N_4 transferred by electrochemical bubbling (a) and wet-etching (b) methods.

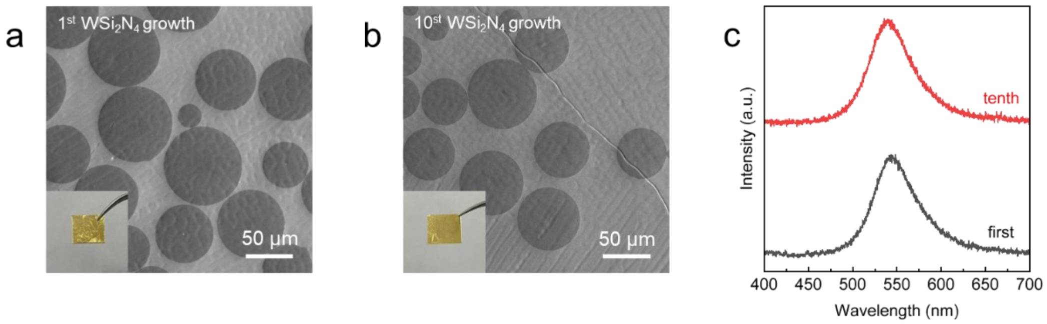

Figure S13. Reuse of Au/W substrate for monolayer WSi_2N_4 growth. (a, b) SEM images of monolayer WSi_2N_4 domains grown on the original Au/W substrate (a) and the reused Au/W substrate for 10 times (b). The insets in (a) and (b) show the photographs of the original Au/W substrate and reused Au/W substrate for 10 times, respectively. (c) Corresponding PL spectra of the WSi_2N_4 monolayer in (a) and (b), confirming that the crystal quality of monolayer WSi_2N_4 grown on the reused Au/W substrate is the same as that of those grown on the original one.

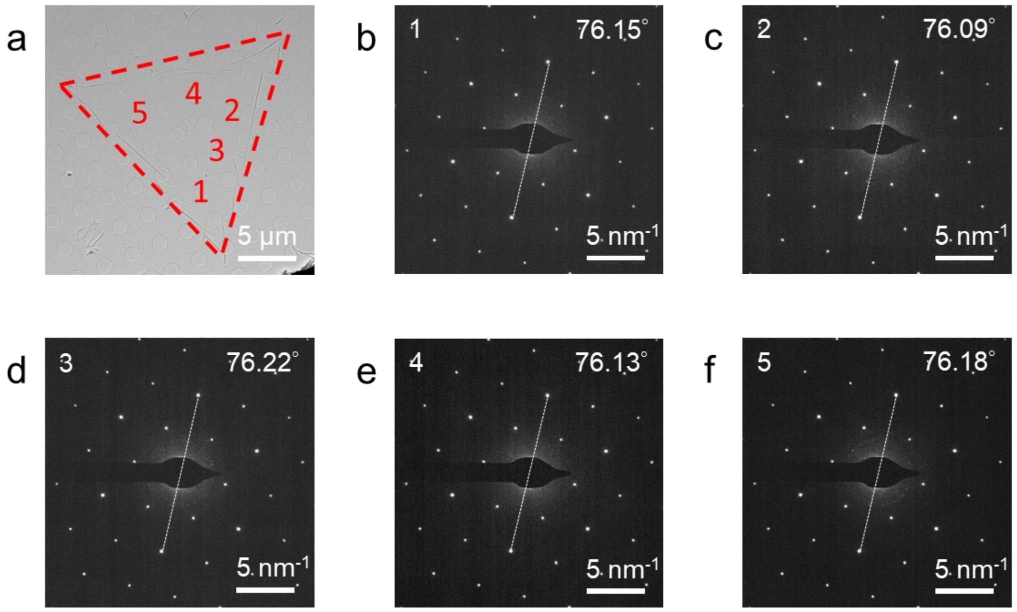

Figure S14. TEM characterizations of a triangular monolayer WSi_2N_4 domain. (a) low-magnification TEM image of a triangular monolayer WSi_2N_4 domain. (b-f) Corresponding SAED patterns taken from the five regions marked by numbers in (a), showing the same crystalline orientation. The dashed lines represent the rotation angles (76.15, 76.09, 76.22, 76.13 and 76.18 °) relative to the horizontal line.

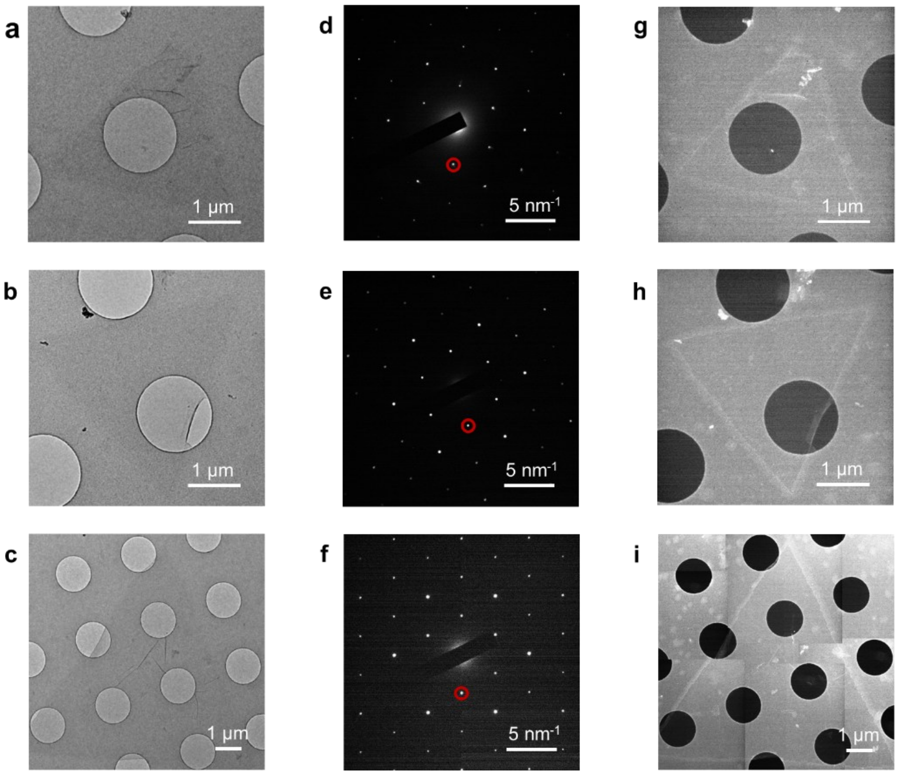

Figure S15. TEM characterization of triangular monolayer WSi_2N_4 domains. (a-c) Low-magnification STEM images of triangular monolayer WSi_2N_4 domains. (d-f) Corresponding SAED patterns. (g-i) Dark-field TEM images of the domains obtained based on the specific diffraction spots indicated by red circles in (d–f).

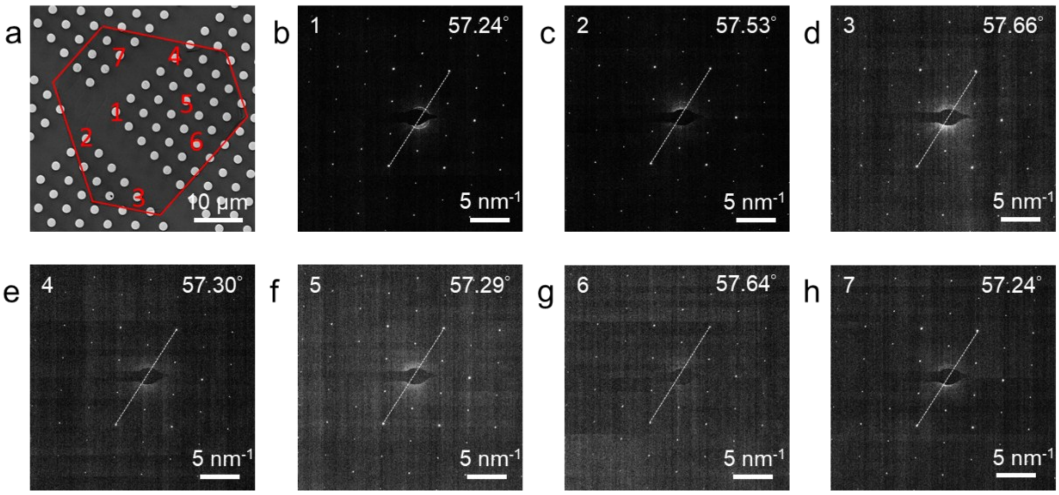

Figure S16. TEM characterizations of a hexagonal monolayer WSi_2N_4 domain. (a) low-magnification TEM image of a hexagonal monolayer WSi_2N_4 domain. (b-h) Corresponding SAED patterns taken from the seven regions marked by numbers in (a), showing the same crystalline orientation. The dashed lines represent the rotation angles (57.24, 57.53, 57.66, 57.30, 57.29, 57.64 and 57.24 °) relative to the horizontal line.

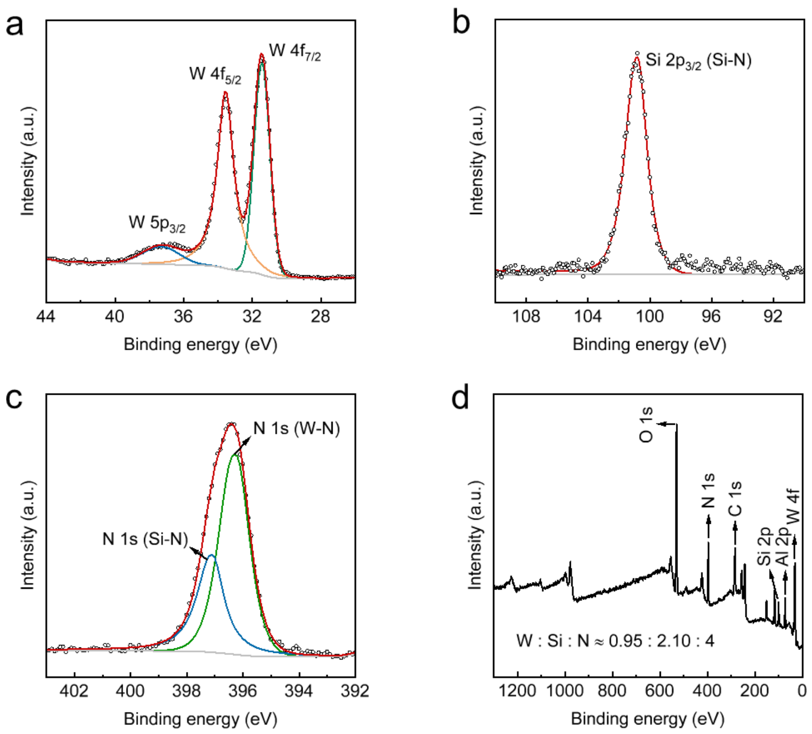

Figure S17. W 4f (a), Si 2p (b), N 1s (c) and survey (d) XPS spectra of a monolayer WSi_2N_4 film transferred onto a sapphire substrate, confirming a W: Si: N atomic ratio of 0.95: 2.10: 4.

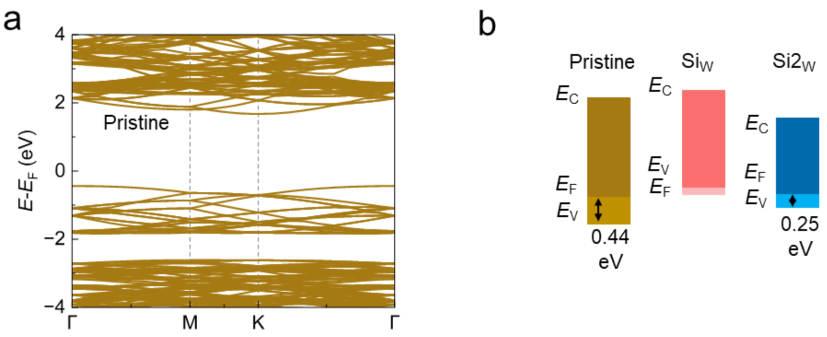

Figure S18. Electronic properties of pristine and defective monolayer WSi_2N_4. (a) Calculated electronic band structures of pristine 5 × 5 × 1 monolayer WSi_2N_4 supercell. (b) Band alignment of pristine and defective monolayer WSi_2N_4.

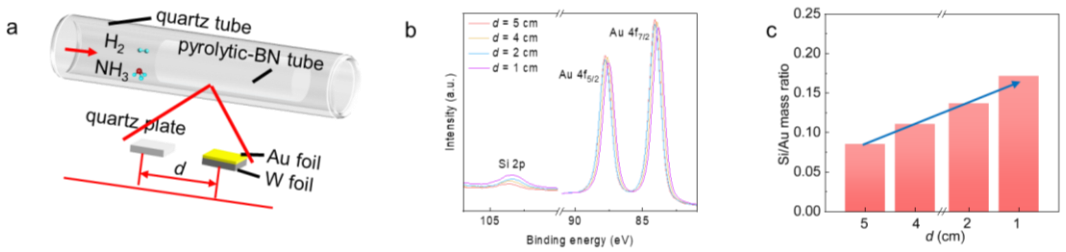

Figure S19. Controlling Si source concentration during WSi_2N_4 growth. (a) Schematic diagram of the growth setup with adjustable quartz plate-to-substrate distance (*d*). (b) XPS spectra (Si 2p and Au 4f region) of the Au/W substrate obtained at different *d*. (c) Quantitative Si content on the Au/W substrate derived from XPS as a function of *d*. The results clearly show that the silicon source concentration increases as *d* decreases.

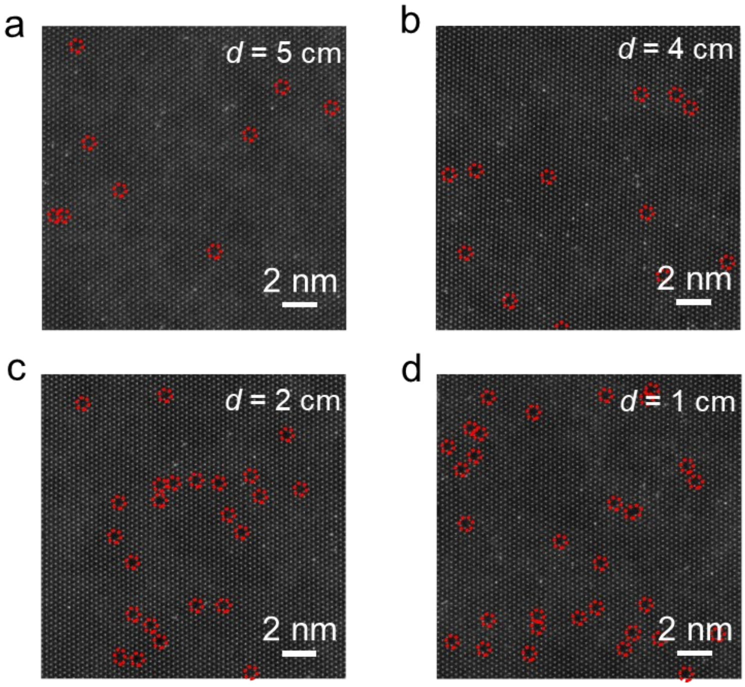

Figure S20. HAADF-STEM images of monolayer WSi_2N_4 grown at different distances (*d*) between the quartz plate and the growth substrate. (a) *d* = 5 cm. (b) *d* = 4 cm. (c) *d* = 2 cm. (d) *d* = 1 cm.

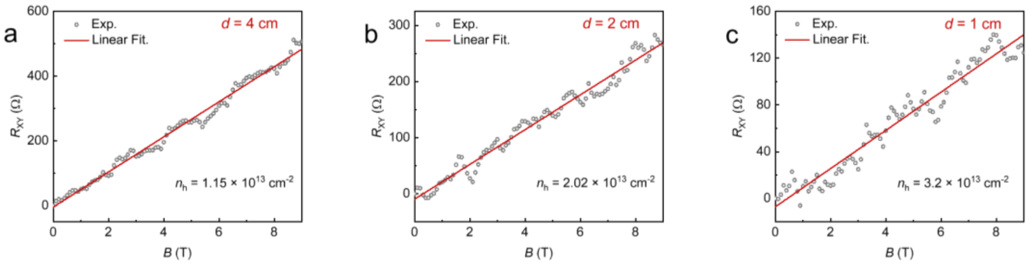

Figure S21. Carrier concentration determined from Hall measurements of monolayer WSi_2N_4 grown at different distances (*d*) between the quartz plate and the growth substrate. (a) $d = 4$ cm. (b) $d = 2$ cm. (c) $d = 1$ cm.

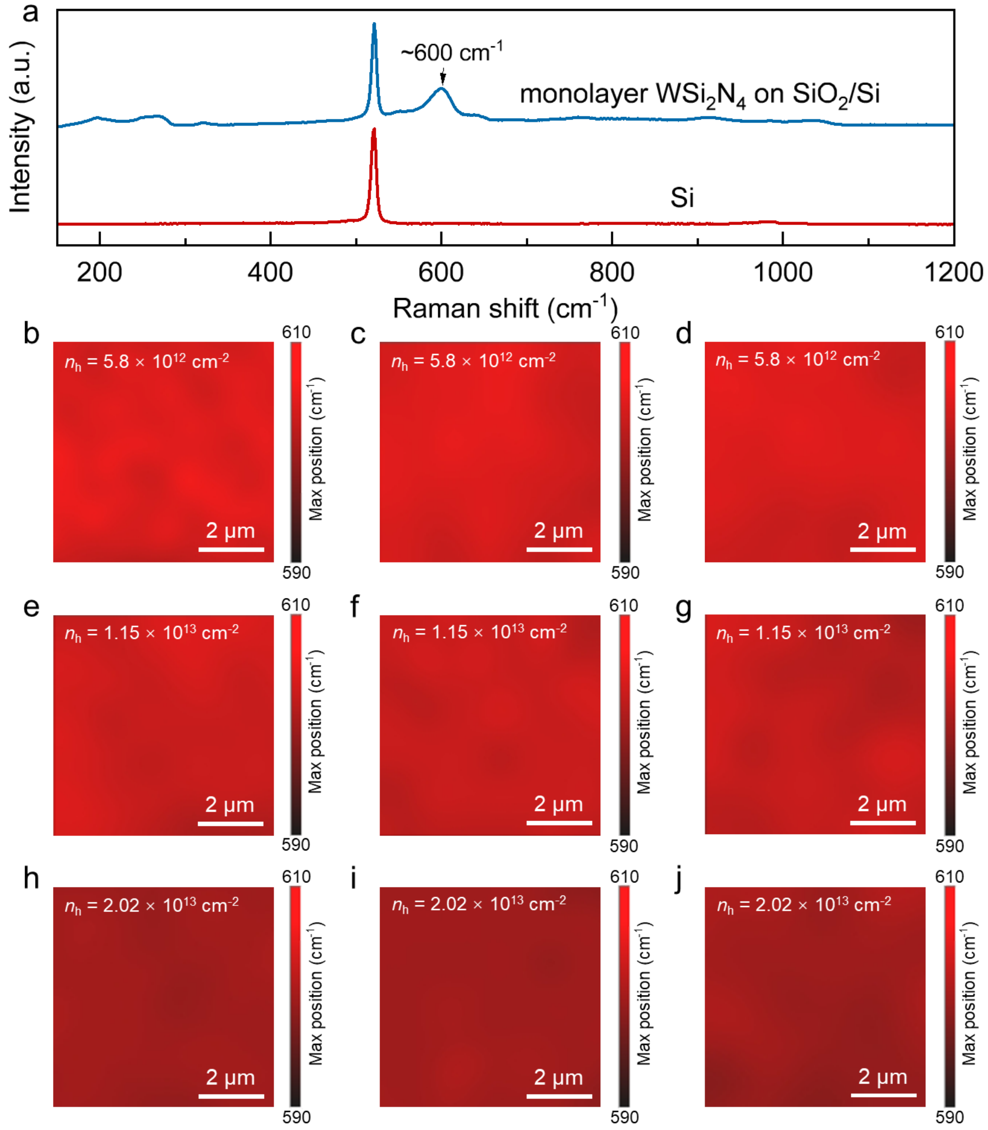

Figure S22 (a) Raman spectra of monolayer WSi_2N_4 measured under an excitation wavelength of 325 nm. (b-j) Raman peak position mappings of monolayer WSi_2N_4 with varying hole concentration (n_h).

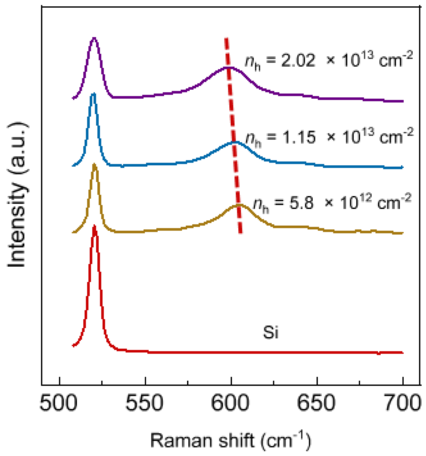

Figure S23 Raman spectra of monolayer WSi_2N_4 with varying hole concentration (n_h).

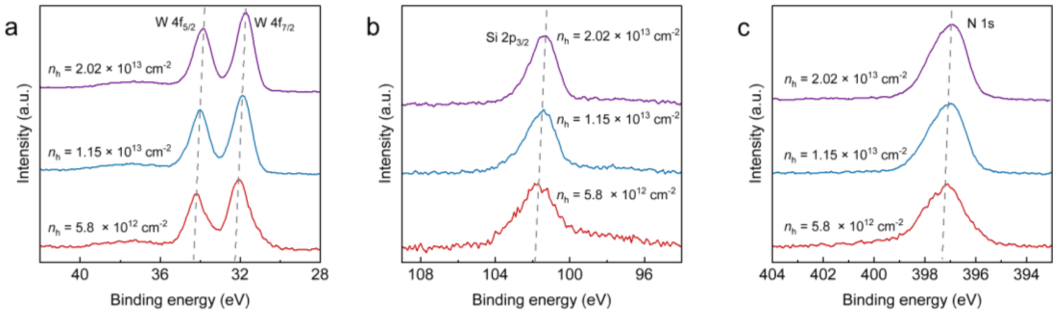

Figure S24. XPS spectra of W 4f (a), Si 2p (b), and N 1s (c) in monolayer WSi_2N_4 with varying hole concentrations (n_h). Notably, as the hole concentration increases from 5.8 × 10^{12} cm^{-2} to 2.02 × 10^{13} cm^{-2}, the XPS peaks of monolayer WSi_2N_4 exhibit a systematic red shift: the W $4f_{7/2}$ peak shifts from 34.2 eV to 33.8 eV, the W $4f_{5/2}$ peak from 32.1 eV to 31.7 eV, the Si 2p peak from 101.7 eV to 101.3 eV, and the N 1s peak from 397.2 eV to 397.0 eV.

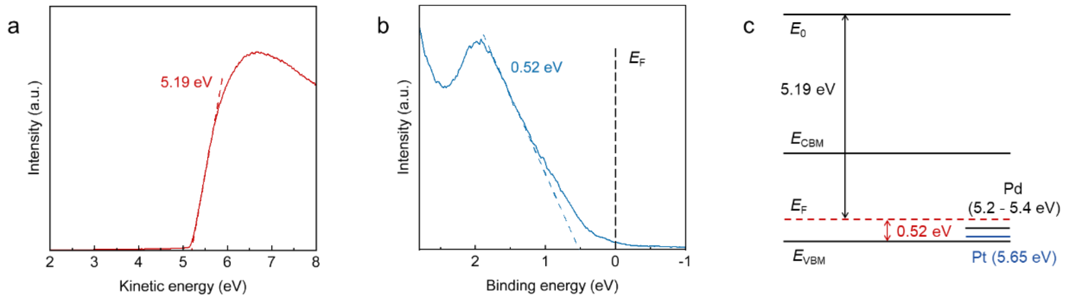

Figure S25. Ultraviolet photoelectron spectroscopy (UPS) spectra and the derived energy band diagram of monolayer WSi_2N_4. (a) Secondary electron cutoff region of the UPS spectrum. Values indicate the work function of monolayer WSi_2N_4. (b) Valence region relative to the Fermi level (E_F) in the UPS spectrum. (c) Energy band diagram of monolayer WSi_2N_4.

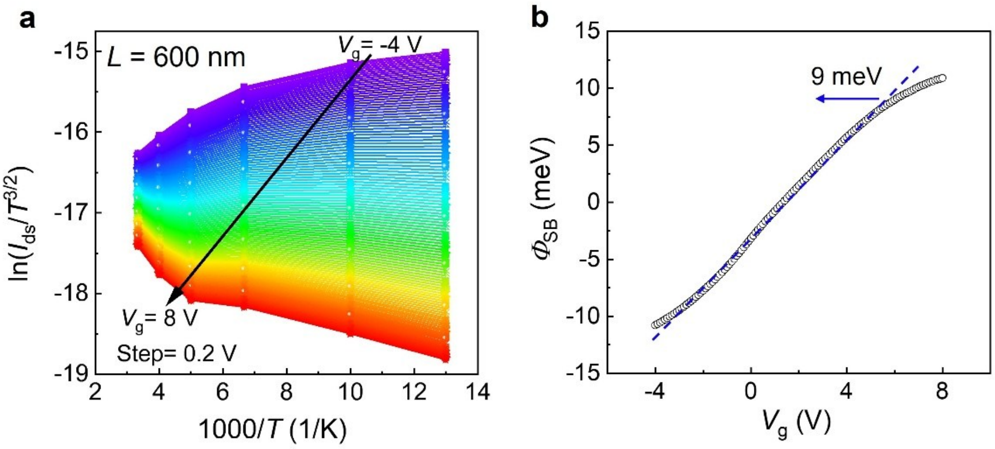

Figure S26 (a) Arrhenius plots of the monolayer WSi_2N_4 FETs on various V_g. (b) The extracted Schottky barrier heights of the monolayer WSi_2N_4 FETs.

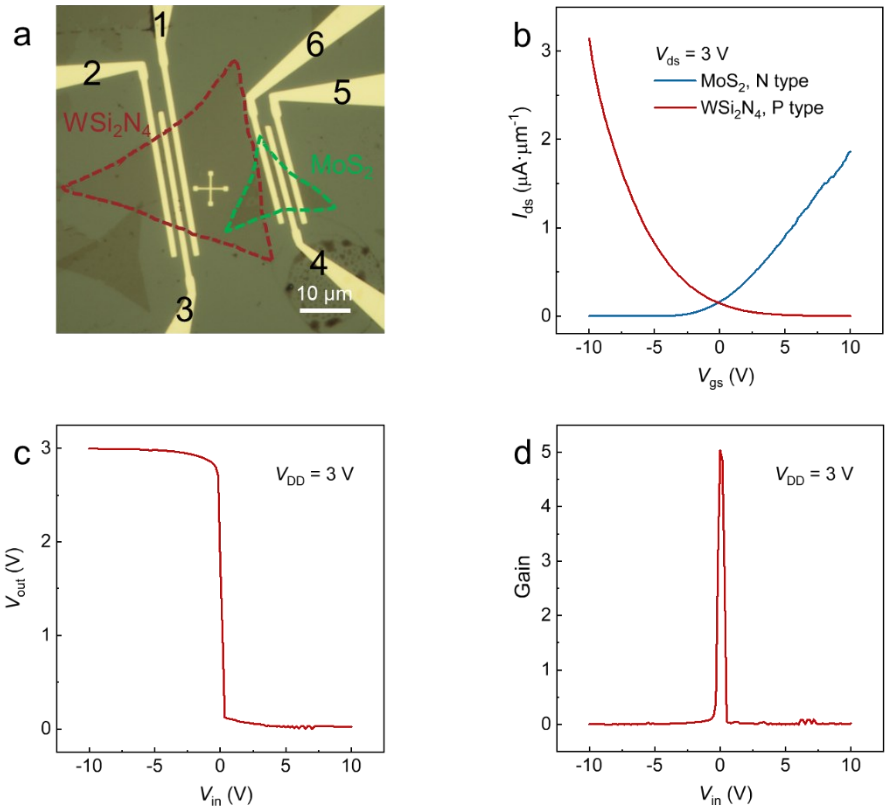

Figure S27. 2D CMOS inverter constructed by p-type monolayer WSi_2N_4 and n-type monolayer MoS_2. (a) Optical image of the 2D inverter. (b) Transfer characteristics of p-type monolayer WSi_2N_4 and n-type monolayer MoS_2 FETs in a CMOS inverter, respectively. (c) Voltage transfer curve of 2D inverter at $V_{DD} = 3$ V. (d) Extracted voltage gain from voltage transfer curve, indicating that the voltage gain ($-d(V_{out})/d(V_{in})$) of the inverter reaches 5 at $V_{DD} = 3$ V.

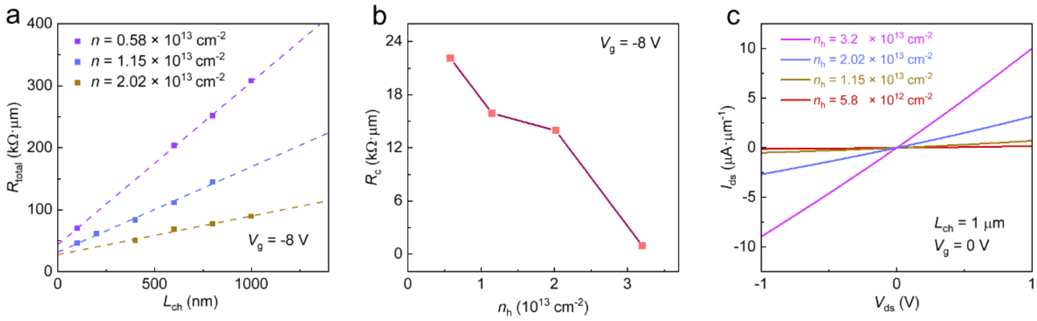

Figure S28. Contact resistance (a, b) and output characteristics (c) of monolayer WSi_2N_4 FETs with varying hole concentrations.

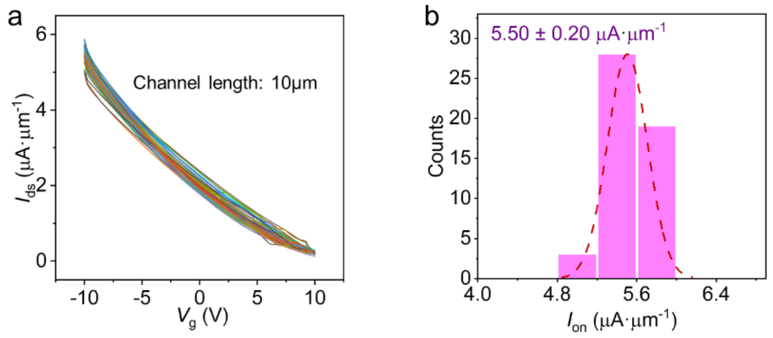

Figure S29. Electrical characterizations of the FET devices fabricated with a monolayer WSi_2N_4 film. (a) Transfer characteristics of 50 WSi_2N_4 FETs with 10-μm channel length. (b) Statistical distributions of on-state current density.

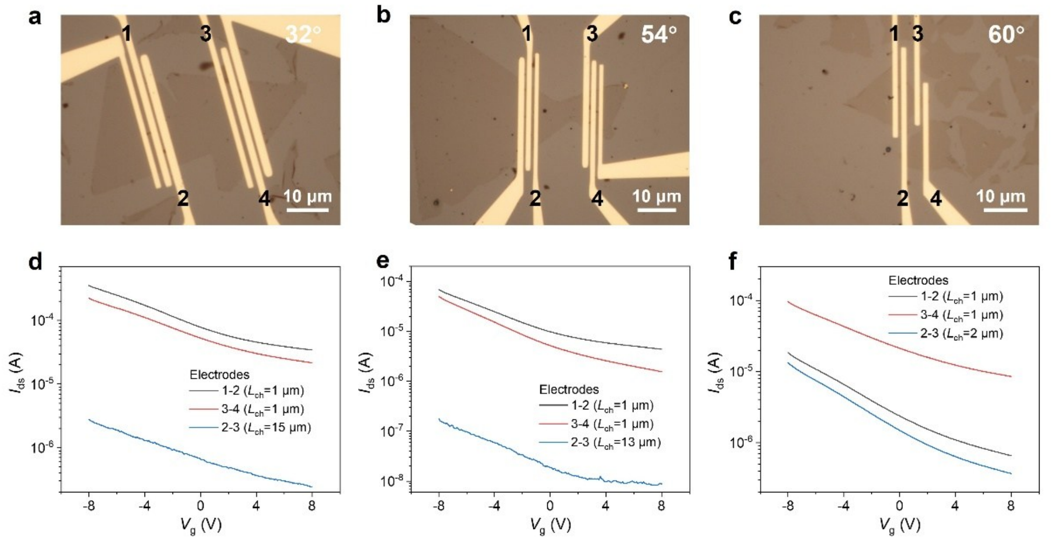

Figure S30. The electrical performance of WSi_2N_4 single-crystal and poly-crystal FETs. (a-c) The optical microscopy images of WSi_2N_4 devices with various grain boundary angles. The numbers label the electrode pair used in electrical measurements. The electrode 1-2 and 3-4 are channels of single-crystal, while electrode 2-3 is channel contacting grain boundary. (d-f) The corresponding transfer curves of WSi_2N_4 FET devices.

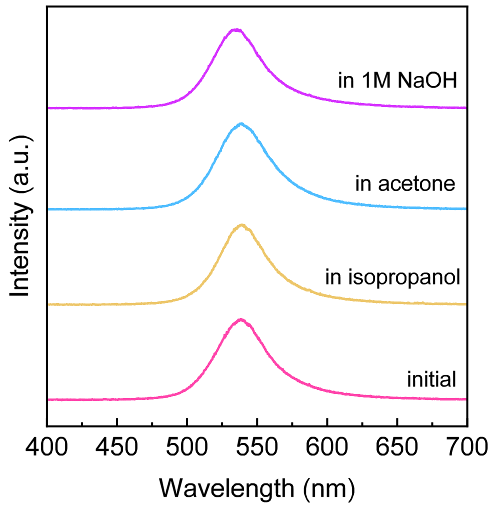

Figure S31. PL spectra of a monolayer WSi_2N_4 domain transferred onto a SiO_2/Si substrate after immersing in various solvents for 24 h, including 1 M NaOH solution, acetone and isopropanol, which are the solvents used in the bubbling transfer process. The unchanged PL spectra confirm that these solvents do not damage the monolayer WSi_2N_4 crystals.

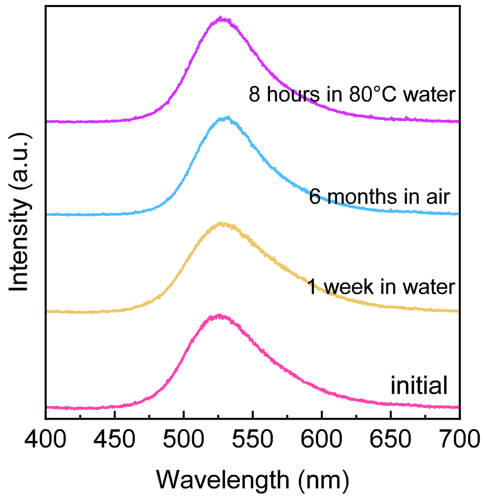

Figure S32. PL spectra of a monolayer WSi_2N_4 transferred onto a SiO_2/Si substrate after placing in non-degassed deionized water for 1 week, in air for 6 months, and in 80 °C non-degassed deionized water for 8 h. The unchanged PL spectra demonstrate the outstanding environmental stability of monolayer WSi_2N_4 crystals.

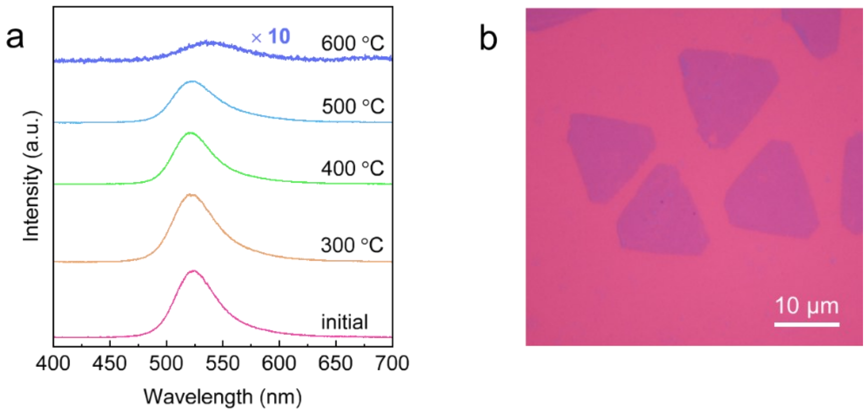

Figure S33. (a) PL spectra of a monolayer WSi_2N_4 transferred onto a SiO_2/Si substrate after annealing at different temperatures under 200 sccm Ar for 3 h. Compared to the original monolayer WSi_2N_4, no discernible change can be observed in the PL spectrum after annealing at 300 °C in Ar for 3 h. Moreover, there still exists a weak PL peak for the sample after annealing at 600 °C in Ar for 3 h, demonstrating the outstanding thermal stability of monolayer WSi_2N_4. (b) Optical image of the monolayer WSi_2N_4 domains after annealing at 600 °C in Ar for 3 h.

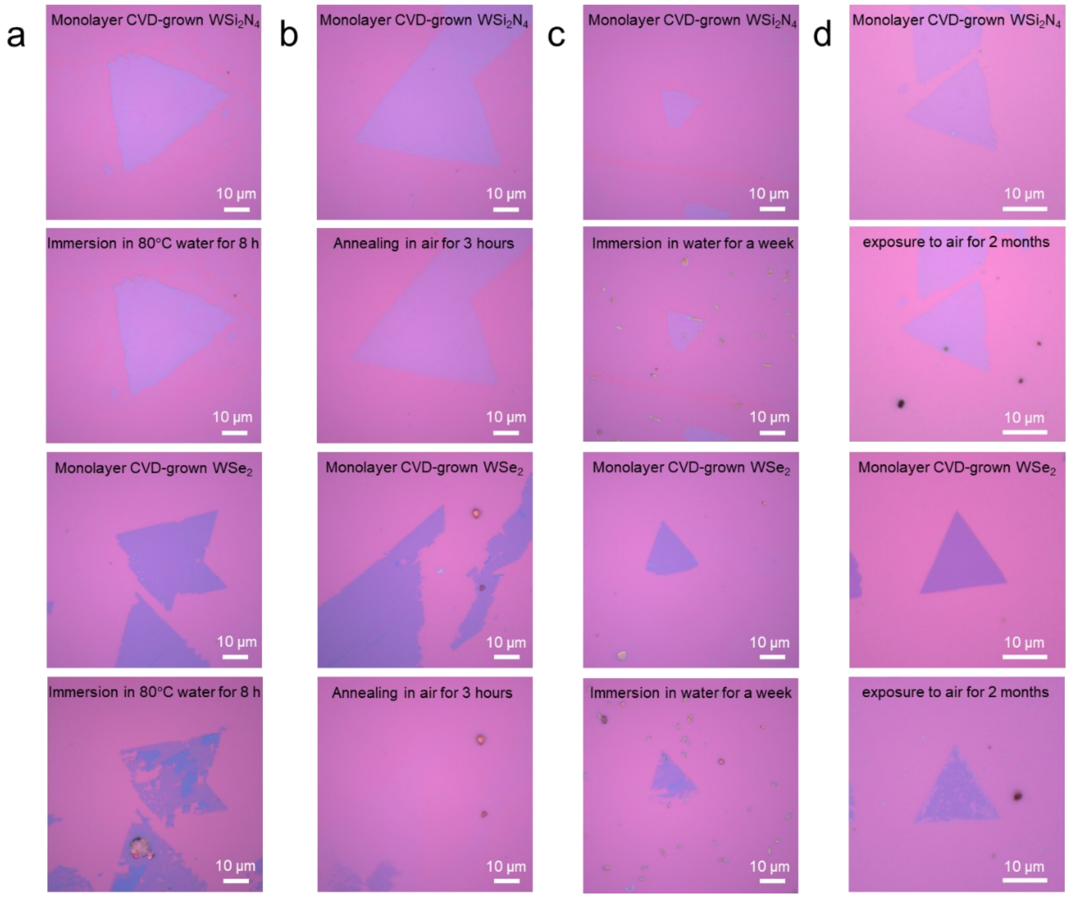

Figure S34. Comparison of stability of monolayer WSi_2N_4 and monolayer WSe_2. (a-d) Optical images of monolayer WSi_2N_4 (upper two panels) and monolayer WSe_2 (lower two panels) before and after treatment in non-degassed deionized water at 80 °C for 8 h (a), in air at 300 °C for 3 h (b), in non-degassed deionized water for 1 week (c), and in air for 2 months (d). Both monolayer WSi_2N_4 and monolayer WSe_2 were grown by CVD.

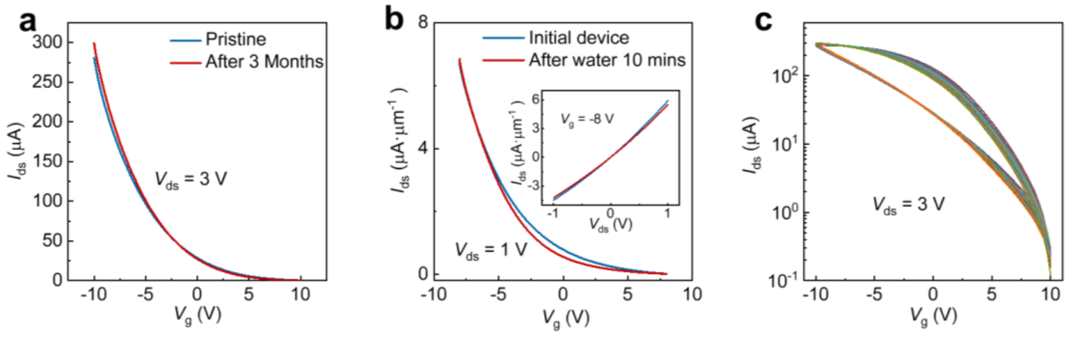

Figure S35. Environmental and operational stability of monolayer WSi_2N_4 FETs. (a) Transfer curves of a monolayer WSi_2N_4 FET before and after 3 months in ambient air. (b) Transfer and output curves of a monolayer WSi_2N_4 FET before and after 10 minutes of immersion in deionized water. (c) Transfer curves of a monolayer WSi_2N_4 device under 22 cycling measurements.

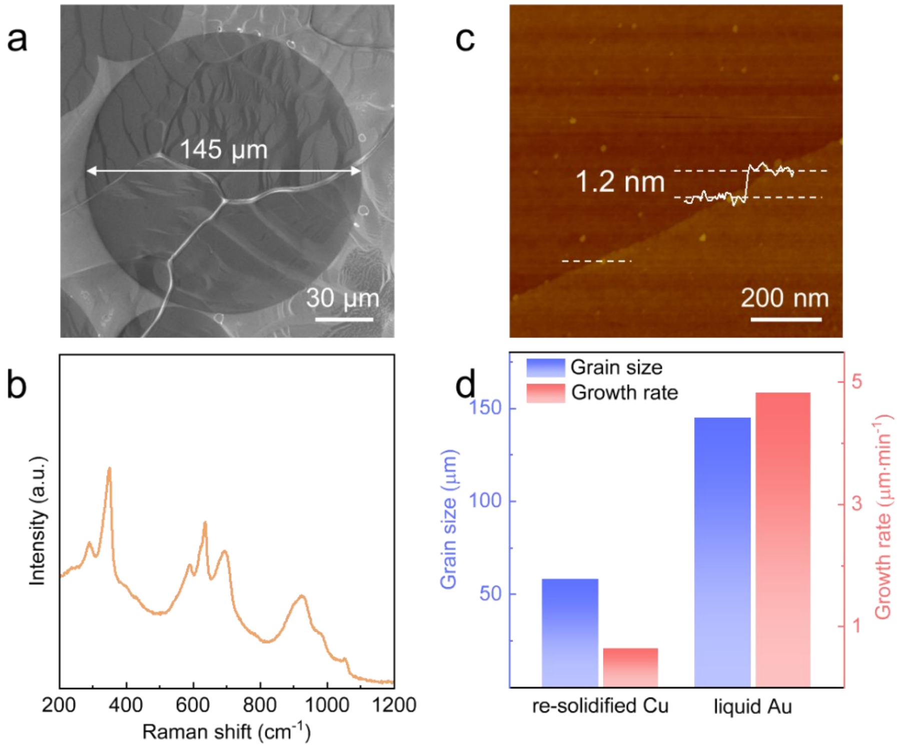

Figure S36. Rapid growth of monolayer $MoSi_2N_4$ crystals. (a) SEM image of a circular $MoSi_2N_4$ domain with a lateral size of ~145 μm. (b) Typical Raman spectrum of a monolayer $MoSi_2N_4$. (c) AFM image of a monolayer $MoSi_2N_4$, showing a thickness of ~1.2 nm. (d) Comparison of the lateral sizes and growth rates of monolayer $MoSi_2N_4$ domains grown with liquid Au/Mo film/Re foil and solid Cu/Mo foil as the growth substrate[7].

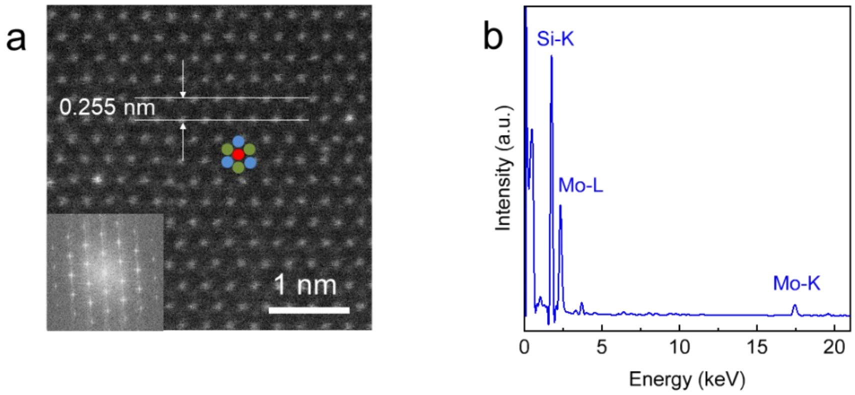

Figure S37. Crystal structure and chemical composition of monolayer $MoSi_2N_4$. (a) Atomic-level HAADF-STEM image of a monolayer $MoSi_2N_4$. The blue, green and red dots mark the Mo, Si and N atoms, respectively. Inset: The corresponding fast Fourier transform (FFT) pattern. (b) EDS profile of monolayer $MoSi_2N_4$.

Supplementary Table S1. Comparison of Young's modulus and breaking strength of monolayer WSi_2N_4 with those of typical 2D materials.

Materials	Method	Young's modulus (GPa)	Breaking strength (GPa)	Reference
WSi_2N_4	Experiment	538.11 ± 103.50	61.9 ± 17.49	**This Work**
	DFT	506	55.5 (a); 59.2 (z)	*9*
Graphene	Experiment	1014.9 ± 149.3	130 ± 10	*10*
	DFT	1050	110 (a); 121 (z)	*11*
h-BN	Experiment	865 ± 73	70.5 ± 5.5	*12*
	DFT	835.93	70.54 (a); 78.62 (z)	*13*
$MoSi_2N_4$	Experiment	491.4 ± 139.1	65.8 ± 18.3	*7*
	DFT	479.08	48.29 (a); 49.08 (z)	
MoS_2	Experiment	270 ± 100	22 ± 4	*14*
	DFT	210	26.8	*15*
$Nb_4C_3T_x$	Experiment	386 ± 13	26 ± 1.6	*16*
$Ti_3C_2T_x$	Experiment	333 ± 30	17.3 ± 1.6	*17*
Bi_2O_2Se	Experiment	88.7 ± 14.4	19.23 ±3.7	*18*
MoB_2	Experiment	517	11.1	*19*

DFT: Density functional theory; z: Zigzag-edge direction; a: Armchair direction.

Supplementary Table S2. Comparison of the Schottky barrier heights of monolayer WSi_2N_4 FETs with the reported p-type semiconductors-based FETs.

**Materials**	**Contact metal**	**Schottky barrier height (meV)**	**Reference**
1L WSi_2N_4	Pt contact	9	**This work**
1L WSe_2	vdW Pd	-3	*20*
	Pt contact	400	*21*
ML WSe_2	Mixed-Dimensional Contact	90	*22*
1L Nb-doped WSe_2	$NbSe_2$ contact	-152	*23*
1L Nb-doped MoS_2	NbS_2 contact	230	*24*
ML $MoTe_2$	vdW Au/1T′	-14	*25*
	$PdTe_2$ contact	65	*26*
	Gr/Pd Contact	69	*27*
β-Bi_2O_3 (nonlayered)	Au contact	-5.19	*28*
α-MnS (nonlayered)	Cr contact	70.8	*29*

Supplementary Table S3. Benchmarking table for the monolayer WSi_2N_4-based FETs.

Carrier concentration (cm^{-2})	Contact metal	Channel length (μm)	Bottom gate dielectrics	R_c (kΩ μm)	I_{on}/I_{off} ratio	μ_h ($cm^2 V^{-1} s^{-1}$)	I_{on} at V_{ds} = -1 V (μA μm^{-1})
5.8 × 10^{12}	Pd/Au	1	30 nm-HfO_2	22.10	500	5.49	2.65
5.8 × 10^{12}	Pd/Au	1	30 nm-HfO_2	22.10	300	4.90	2.67
5.8× 10^{12}	Pd/Au	1	30 nm-HfO_2	22.10	340	4.90	2.39
1.15 × 10^{13}	Pd/Au	1	30 nm-HfO_2	15.91	50	2.40	3.27
1.15 × 10^{13}	Pd/Au	1	30 nm-HfO_2	15.91	60	6.94	5.39
1.15 × 10^{13}	Pd/Au	1	30 nm-HfO_2	15.91	40	9.50	7.83
2.02 × 10^{13}	Pd/Au	1	30 nm-HfO_2	13.94	11	10.76	10.76
2.02 × 10^{13}	Pd/Au	1	30 nm-HfO_2	13.94	8	8.03	10.54
2.02 × 10^{13}	Pd/Au	1	30 nm-HfO_2	13.94	9	6.26	8.23
3.2 × 10^{13}	Pd/Au	1	30 nm-HfO_2	0.95	7	9.67	13.93
3.2 × 10^{13}	Pd/Au	1	30 nm-HfO_2	0.95	8	12.92	22.12
3.2 × 10^{13}	Pd/Au	1	30 nm-HfO_2	0.95	5	5.54	11.25
3.2 × 10^{13}	Pd/Au	1	30 nm-HfO_2	0.95	6	10.76	27.07
3.2 × 10^{13}	Pd/Au	0.8	30 nm-HfO_2	0.95	6	9.89	31.95
3.2 × 10^{13}	Pd/Au	0.6	30 nm-HfO_2	0.95	7	10.02	41.91
3.2 × 10^{13}	Pd/Au	0.4	30 nm-HfO_2	0.95	6	10.36	63.71

3.2×10^{13}	Pd/Au	0.2	30 nm-HfO_2	0.95	5	7.57	111.22
3.2×10^{13}	Pd/Au	0.1	30 nm-HfO_2	0.95	5	4.72	149.19
3.2×10^{13}	Pt/Au	2.63	30 nm-HfO_2	1.29	4.1	4.63	8.08
3.2×10^{13}	Pt/Au	1.61	30 nm-HfO_2	1.29	3.2	5.26	15.18
3.2×10^{13}	Pt/Au	0.63	30 nm-HfO_2	1.29	4.7	6.09	26.9
3.2×10^{13}	Pt/Au	0.56	30 nm-HfO_2	1.29	3	3.18	37.14
3.2×10^{13}	Pt/Au	0.18	30 nm-HfO_2	1.29	3.2	3.67	78.32

Supplementary Table S4. Comparison of the on-state current densities of monolayer WSi_2N_4 FETs with the reported p-type monolayer semiconductors-based FETs.

**Materials**	**Contact or doping technique**	**Channel length *L* (μm)**	**Drain voltage V_{ds} (V)**	**On-state current density I_{on} (μA μm^{-1})**	**Reference**
WSi_2N_4	Pd contact	0.1	1	150	**This Work**
		0.2	1	111.2	
		0.4	1	63.7	
		0.6	1	41.9	
		0.8	1	31.9	
		1.0	1	27	
WSi_2N_4	Pt contact	0.18	1	78.32	
		0.56	1	37.14	
		0.63	1	26.9	
WSe_2	Pt contact	0.7	1	70.17	*30*
WSe_2	Clean vdW Pt contact	1.5	1	7.6	*21*
WSe_2	vdW Pd contact	0.1	1	105	*20*
		2	1	18	
		4	1	13	
		6	1	10	
		8	1	8.5	
		10	1	6.5	
WSe_2	α-$RuCl_3$ contact	0.5	1	31	*31*
WSe_2	Cr contact	1	2	1.6	*23*
WSe_2	Cr contact	2	2	0.8	

WSe_2	Cr contact	3	2	0.6	
b-As	Au contact	2	2	1.3	*32*
$MoSi_2N_4$	Ti contact	30	1	0.05	*7*
$MoTe_2$	Cr contact	1	1	1.75	*33*
SnS_2	In-Doped	3	1	2.6	*34*
BP	vdW Pt	15.4	1	0.01847	*35*
GeAs	vdW Pt	13.3	1	0.02095	

Supplementary Table S5. Comparison of the contact resistances of monolayer WSi_2N_4 FETs with the reported p-type monolayer semiconductors-based FETs.

**Materials**	**Contact or doping technique**	**Hole concentration n_h (cm^{-2})**	**Contact resistance R_c (kΩ μm)**	**Reference**
WSi_2N_4	Pd contact	3.2×10^{13}	0.95	**This Work**
WSe_2	vdW Pd contact	5×10^{12}	12	*20*
WSe_2	Pd contact	5×10^{12}	90	
WSe_2	α-$RuCl_3$ contact	3.1×10^{13}	4	*31*
WSe_2	α-$RuCl_3$ contact	3.25×10^{13}	1.7	*36*
Nb-doped WSe_2	Cr contact	2.99×10^{11}	10888.78	*23*
Nb-doped WSe_2	Cr contact	6.01×10^{11}	3540.04	
Nb-doped WSe_2	Cr contact	9.05×10^{11}	395.42	
Nb-doped WSe_2	Cr contact	1.379×10^{12}	70.6	
Nb-doped WSe_2	$NbSe_2$ contact	1.379×10^{12}	2.46	

Supplementary Table S6. The effect of grain boundary on the carrier mobility of WSi_2N_4 FETs.

Angle	Mobility-SC (cm^2 V^{-1} s^{-1})	Mobility-GB (cm^2 V^{-1} s^{-1})	Change
32°	8.6	4.8	-44%
54°	3.0	1.4	-53%
60°	5.4	4.8	-11%

Supplementary Table S7. Defect formation energies of WSi_2N_4 and WSe_2.

Configuration	E_{For} (eV/Å^2)	Reference
	WSi_2N_4	
V_{N1}	4.58	**This work**
V_{N2}	5.59	
O_{N1}	0.48	
O_{N2}	0.71	
	WSe_2	
V_{Se}	2.86	**This work**
O_{Se}	-1.29	
V_{Se}	1.85	*37*
O_{Se}	0.05	*37*

We calculated the formation energies of different defects in WSi_2N_4, including N vacancy in inner layer (V_{N1}), N vacancy in outer layer (V_{N2}) and the O substitutions at these sites (O_{N1} and O_{N2}). For comparison, we also calculated the formation energies of Se vacancy (V_{Se}) and O substitution in Se site in WSe_2 (O_{Se}). Here, the supercell is 4 × 4 × 1 for both WSi_2N_4 and WSe_2. The calculated results are summarized in Table S7.

It can be found that the formation energies of different defects in WSi_2N_4 follow the order: $V_{N2} > V_{N1} > O_{N2} > O_{N1}$. We can confirm that the outer nitrogen layer shows higher stability than the inner one, which is consistent the fact that the outer Si-N layer has stronger covalent bonding[38]. In contrast, both V_{Se} and O_{Se} defects in WSe_2 exhibit much lower formation energies compared to those in WSi_2N_4, suggesting that WSe_2 is more susceptible to vacancy formation and oxygen doping. These results support that WSi_2N_4 is more stable than WSe_2. Generally, in actual scenarios, O_2 or H_2O can only contact the outer N layer; therefore, the strong covalent bonding of the outer Si-N layer

plays a crucial role in protecting WSi_2N_4 from degradation and oxidation.

First-principles calculations were performed by using the Vienna ab initio simulation package (VASP) based on DFT. The projected augmented wave (PAW) method and generalized gradient approximation with the Perdew-Burke-Ernzerh exchange-correlation functional (GGA-PBE) and the DFT-D3 method (Grimme scheme with zero damping) were used. The plane-wave energy cutoff of 500 eV was adopted for all calculations. A large vacuum space was set to 20 Å to avoid the interactions between the neighboring layers. The monolayer WSi_2N_4, WSe_2, O_2, N_2 and Se_{32} were relaxed until the energies and forces converged below 10^{-8} eV and 0.02 eV Å^{-1}, respectively. For defect calculations, 4 × 4 × 1 supercells were constructed, containing four types of defects in WSi_2N_4 (V_{N1}, V_{N2}, O_{N1}, and O_{N2}) and two types in WSe_2 (V_{Se} and O_{Se}). These supercells were relaxed with convergence thresholds of 10^{-6} eV for energy and 0.02 eV·Å^{-1} for forces. The k-point mesh of 15 × 15 ×1 was used for pristine monolayers, while a 2 × 2 × 1 mesh was applied for defect supercells.

Supplementary references

1 Kresse G and Furthmuller J. Efficiency of ab-initio total energy calculations for metals and semiconductors using a plane-wave basis set. *Comput Mater Sci* 1996; **6**: 15-50.

2 Kresse G and Furthmuller J. Efficient iterative schemes for ab initio total-energy calculations using a plane-wave basis set. *Phys Rev B* 1996; **54**: 11169-86.

3 Hafner J. Materials simulations using VASP - a quantum perspective to materials science. *Comput. Phys Commun* 2007; **177**: 6-13.

4 Blöchl PE. Projector augmented-wave method. *Phys Rev B* 1994; **50**: 17953-79.

5 Perdew JP, Burke K, Ernzerhof M. Generalized gradient approximation made simple. *Phys Rev Lett* 1996; **77**: 3865-8.

6 Kohn W and Sham, LJ. Self-consistent equations including exchange and correlation effects. *Phys Rev* 1965; **140**: A 1133-8.

7 Hong Y-L, Liu ZB, Wang L *et al.* Chemical vapor deposition of layered two-dimensional $MoSi_2N_4$ materials. *Science* 2020; **369**: 670-4.

8 Grimme S, Antony J, Ehrlich S *et al.* A consistent and accurate ab initio parametrization of density functional dispersion correction (DFT-D) for the 94 elements H-Pu. *J Chem Phys* 2010; **132**: 154104.

9 Mortazavi B, Javvaji B, Shojaei F *et al*. Exceptional piezoelectricity, high thermal conductivity and stiffness and promising photocatalysis in two-dimensional $MoSi_2N_4$ family confirmed by first-principles. *Nano Energy* 2021; **82**: 105716.

10 Lee C, Wei XD, Kysar JW *et al*. Measurement of the elastic properties and intrinsic

strength of monolayer graphene. *Science* 2008; **321**: 385-8.

11 Liu F, Ming PB, Li J. Ab initio calculation of ideal strength and phonon instability of graphene under tension. *Phys Rev B* 2007; **76**: 064120.

12 Falin A, Cai QR, Santos EJG *et al*. Mechanical properties of atomically thin boron nitride and the role of interlayer interactions. *Nat Commun* 2017; **8**: 15815.

13 Peng Q, Ji W, De S. Mechanical properties of the hexagonal boron nitride monolayer: ab initio study. *Comput Mater Sci* 2012; **56**: 11-7.

14 Bertolazzi S, Brivio J, Kis A. Stretching and breaking of ultrathin MoS_2. *ACS Nano* 2011; **5**: 9703-9.

15 Cooper RC, Lee C, Marianetti CA *et al*. Nonlinear elastic behavior of two-dimensional molybdenum disulfide. *Phys Rev B* 2013; **87**: 035423.

16 Lipatov A, Alhabeb M, Lu HD *et al*. Electrical and elastic properties of individual single-layer $Nb_4C_3T_x$ MXene flakes. *Adv Electron Mater* 2020; **6**: 1901382.

17 Lipatov A, Lu HD, Alhabeb M *et al*. Elastic properties of 2D $Ti_3C_2T_X$ MXene monolayers and bilayers. *Sci Adv* 2018; **4**: eaat0491.

18 Chen WJ, Khan U, Feng SM *et al*. High-fidelity transfer of 2D Bi_2O_2Se and its mechanical properties. *Adv Funct Mater* 2020; **30**: 2004960.

19 Si JJ, Yu JQ, Lan HH *et al*. Chemical potential-modulated ultrahigh-phase-purity growth of ultrathin transition-metal boride single crystals. *J Am Chem Soc* 2023; **145**: 3994-4002.

20 Li MM, Zhang XY, Zhang ZM *et al*. Unipolar p-type monolayer WSe_2 field-effect transistors with high current density and low contact resistance enabled by van der

Waals contacts. *Nano Res* 2024; **17**: 10162-9.

21 Wang Y, Kim JC, Li Y *et al*. P-type electrical contacts for 2D transition-metal dichalcogenides. *Nature* 2022; **610**: 61-6.

22 Lee S, Lee D, Lee D *et al.* Mixed-dimensional contact architecture to WSe_2 for efficient hole injection. *ACS Appl Mater Interfaces* 2025; **17**: 25500-6.

23 Vu VT, Vu TTH, Phan TL *et al*. One-step synthesis of $NbSe_2$/Nb-Doped-WSe_2 metal/doped-semiconductor van der Waals heterostructures for doping controlled ohmic contact. *ACS Nano* 2021; **15**: 13031-40.

24 Wang ZY, Tripathi M, Golsanamlou Z *et al.* Substitutional p-type doping in NbS_2-MoS_2 lateral heterostructures grown by MOCVD. *Adv Mater* 2023; **35**: 2209371.

25 Song S, Yoon A, Jang S *et al*. Fabrication of p-type 2D single-crystalline transistor arrays with Fermi-level-tuned van der Waals semimetal electrodes. *Nat Commun* 2023; **14**: 4747.

26 Zheng JY, Miao TT, Xu R *et al.* Chemical synthesis and integration of highly conductive $PdTe_2$ with low-dimensional semiconductors for p-type transistors with low contact barriers. *Adv Mater* 2021; **33**: e2101150.

27 Qi DY, Li P, Ou HH *et al*. Graphene-enhanced metal transfer printing for strong van der Waals contacts between 3D metals and 2D semiconductors. *Adv Funct Mater* 2023; **33**: 2301704.

28 Xiong YH, Xu D, Zou YS *et al*. Vapour–liquid–solid–solid growth of two-dimensional non-layered β-Bi_2O_3 crystals with high hole mobility. *Nat Mater* 2025; **24**: 688-97.

29 Li NN, Zhang Y, Cheng RQ *et al*. Synthesis and optoelectronic applications of a stable p-type 2D material: α-MnS. *ACS Nano* 2019; **13**: 12662-70.

30 Kim KS, Lee D, Chang CS *et al*. Non-epitaxial single-crystal 2D material growth by geometric confinement. *Nature* 2023; **614**: 88-94.

31 Xie JX, Zhang ZC, Zhang HD, *et al*. Low resistance contact to p-type monolayer WSe_2. *Nano Lett* 2024; **24**: 5937-43.

32 Zhong MZ, Xia QL, Pan Lf *et al*. Thickness-dependent carrier transport characteristics of a new 2D elemental semiconductor: black arsenic. *Adv Funct Mater* 2018; **28**: 1802581.

33 Kim C, Moon I, Lee D *et al*. Fermi level pinning at electrical metal contacts of monolayer molybdenum dichalcogenides. *ACS Nano* 2017; **11**: 1588-96.

34 Li ZC, Shu WN, Li QQ *et al*. Nondegenerate p-type In-doped SnS_2 monolayer transistor. *Adv Electron Mater* 2021; **7**: 2001168.

35 Li WY, Tao QY, Li ZW *et al*. Monolayer black phosphorus and germanium arsenide transistors via van der Waals channel thinning. *Nat Electron* 2024; **7**: 131-7.

36 Pack J, Guo YJ, Liu ZY *et al*. Charge-transfer contacts for the measurement of correlated states in high-mobility WSe_2. *Nat Nanotechnol* 2024; **19**: 948-54.

37 Zhang Y-Z, Zhu G-J, Yang J-H. Origin of p-type conductivity in a WSe_2 monolayer. *Nanoscale* 2023; **15**: 12116-22.

38 Liu ZB, Wang L, Hong Y-L *et al*. Two-dimensional superconducting $MoSi_2N_4(MoN)_{4n}$ homologous compounds. *Natl Sci Rev* 2023; 10: nwac273.
